# What’s hidden below definiteness and genitive: on indefinite partitive articles in Romance

**DOI:** 10.1515/ling-2022-0059

**Published:** 2024-04-18

**Authors:** Francesco Pinzin

**Affiliations:** Goethe Universität Frankfurt am Main, Frankfurt am Main, Germany

**Keywords:** Romance, indefinites, partitive articles, definite articles, nanosyntax

## Abstract

In French, Italian, and other Romance languages indefinite nominal phrases can be introduced by what appears to be the conflation of a genitive preposition and a definite article, the so-called “indefinite partitive articles” (e.g., Fr. *Je cuisine de la soupe depuis deux jours*. ‘I’ve been cooking soup for two days’). This is rather unexpected, since these nominal phrases are neither definite nor in a syntactic position in which we expect to find a genitive preposition. This led part of the literature to consider them as built by lexical items synchronically distinct from the genitive preposition/definite article but homophonous with them. This contribution shows how a constituent-based approach to the lexicon-syntax interface as nanosyntax, paired with a specific take on the sequence of syntactic functions, can capture their apparently conflicting distribution without stipulating multiple homophonous lexical items. The key factor in this proposal is a revised analysis of the Romance lexical item (LI) for (i) definite articles – linked to a constituent containing not only features of definiteness but also lower indefinite features and higher nominative/accusative case features – and (ii) the genitive preposition *DE* – linked to a constituent containing not only genitive features but also lower nominative/accusative features. Holding these LIs crosslinguistically stable, the variation attested in this domain is modeled as depending on the amount of functional structure lexicalized by the nominal root in the different languages.

## 1 Introduction

Indefinites in Romance languages have raised substantial interest in the last few decades, especially regarding their crosslinguistic variation (Cardinaletti and Giusti 2016; Delfitto and Schroter 1991; Espinal and Cyrino 2021, 2022; Stark 2008a, 2008b, 2016) and their morphosyntax (Chierchia 1997; Zamparelli 2008). As for the first point – the crosslinguistic variation – it has been observed that the same indefinite context is marked differently in Italian and French. While in Italian (1) the noun *minestra* ‘soup’ is not introduced by any overt functional element (i.e., the noun is bare, *B[are] N[oun]* henceforth), in French (2) we find the overt marker *de la*, literally ‘of the’.

(1)

**Table:** 

*Cucino*	*minestra*	*da*	*due*	*giorni.* (It.)
cook.1sg	soup	from	two	days
‘I have been cooking soup for two days.’

(2)

**Table:** 

*Je*	*cuisine*	*de*	*la*	*soupe*	*depuis*	*deux*	*jours.* (Fr.)
sbj.1sg	cook.1sg	of	the.f.sg	soup	from	two	days
‘I have been cooking soup for two days.’							

In this case, Italian is akin to English, as the translation shows.1I refer here to the use of BNs in English as existential indefinite expressions (*I see dogs every day*), not to their use as kind-referring expressions (*Dogs are mammals*) (see Carlson 1977). See Longobardi (2001) for a detailed Romance/Germanic comparison, where it is shown that, while Germanic BNs are ambiguous between indefinite existential and kind reading, Romance BNs have the existential reading only (see Dobrovie-Sorin and Laca 1996, 2003 for the same claim). Note that in Italian and other Romance languages, kind-denoting expressions are generally introduced by definite determiners, as shown in (i):

(i)

**Table:** 

*(I)	*cani*	*sono*	*mammiferi.* (It.)
the	dogs	are	mammals
‘Dogs are mammals.’

See also Borik and Espinal (2015) for an analysis of generic versus kind reading in Spanish. In this contribution I focus only on indefinites, leaving aside kind-denoting expressions. However, Italian possesses a form that is parallel to French *de la* ‘of.the’ and that, like its French counterpart in (2), may also be used in indefinite contexts (3):

(3)

**Table:** 

*Ho*	*cucinato*	*della*	*minestra.* (It.)
have.1sg	cooked	of.the.f.sg	soup
‘I cooked some soup.’			

In this respect, the main points of interest are how to model this crosslinguistic variation and what it tells us regarding the structure of indefinites.

A closer look at the indefinite markers in (2) and (3) leads us to the second topic: their morphosyntactic structure. These elements are morphologically complex, being composed of the genitive preposition *de* ‘of’ – i.e., the form used to introduce genitive nominal phrases (see *de* ‘of’ in [4]) – and the definite article – i.e., the form used to introduce nominal phrases referring to maximal or unique entities (see *le* and *la* in [4]).2In this contribution, I adopt the maximality approach (Link 1983; Sharvy 1980, among others) for reasons to be clarified in Section 5.

(4)

**Table:** 

*Le*	*jouet*	*de*	*la*	*fille.* (Fr.)
the.m.sg	toy	of	the.f.sg	girl
‘The toy belonging to the girl.’				

This makes (2)–(3) superficially identical to partitive structures like (5) introducing a subset of a definite set (*partitives* henceforth, see Jackendoff 1968; Selkirk 1977, among others), leading part of the literature to label them “indefinite partitive articles” (*indefinite PAs* henceforth) (Pinzin and Poletto 2022a, 2022b; Stark 2008a, 2008b, 2016, among others).

(5)

**Table:** 

*J’*	*ai*	*mangé*	*une*	*partie*	*de*	*la*	*soupe*	*qu’*	*ils*	*m’*
sbj.1sg	have.1sg	eaten	a	part	of	the.f.sg	soup	that	sbj.3pl	dat.1sg
*ont*	*donnée.* (Fr.)									
have.3pl	given									
‘I ate a part of the soup they gave me.’										

In this respect, the focus is on the relationship between their morphological and semantic composition (see Section 3 for a literature review on the topic). First, a genitive preposition is not expected to introduce a direct argument (subject or object). Second, the presence of a definite determiner seems to clash with the general semantics of these constituents, which does not include any reference to a maximal entity in the discourse, as apparent from (2)–(3).

In this contribution, I put forward a novel proposal regarding both the syntactic structure of indefinite PAs and what regulates their crosslinguistic distribution. The core of the proposal is that both the lexical item (*LI* henceforth) for *de* ‘of’ and for the definite article correspond to complex syntactic structures in the lexicon (in line with nanosyntax; Starke 2009, 2014). More precisely, I suggest that (i) the LI for *de* ‘of’ contains not only the syntactic feature for genitive case, but also the syntactic features for nominative and accusative case (assuming Caha’s 2009, 2013, 2019 Case Containment Hypothesis) and (ii) the LI for the definite article contains, in addition to the features allowing it to refer to maximal entities in the discourse, the features for lexicalizing different layers of indefiniteness. I will show how this proposal, in combination with the nanosyntactic lexicalization algorithm (see Caha 2019; Starke 2018), allows these LIs to surface in the different syntactic contexts highlighted in (1)–(5). As a second step, I show how the crosslinguistic variation across Romance languages in this domain can be modeled as depending on the amount of structure directly lexicalized by the LI that corresponds to the noun in each language, in line with other recent proposals in the literature which bind the variation in the lexicalization patterns to differences in the number of functional heads lexicalized by roots (among others, see Caha et al. 2019; Vanden Wyngaerd et al. 2020 for adjectival morphology in Slavic; Caha et al. 2023 for adjectival/verbal *zero derivation* in English and Czech; Janků 2022 for nominal declensions in Czech; Bertocci and Pinzin 2020 for verbal thematic vowels in Latin; Cortiula 2023 for verbal morphology in Friulian).

Such an analysis has positive consequences on at least two fronts. On the one hand, it reconciles an approach adopting single LIs for *de* ‘of’ and definite articles with much of the data showing that indefinite PAs (2)–(3), prepositional phrases (4), and partitives (5) have different syntactic distributions and behave differently when we submit them to the same range of tests (see Section 3): allowing different syntactic structures to be lexicalized by the same LIs solves this apparent paradox. On the other hand, it enables us to model the attested crosslinguistic variation across different Romance languages purely based on the shape of the LIs without referring to syntactic parameters or other sources of variation (i.e., the syntax of indefinites is kept constant in all languages analyzed), in line with the Borer-Chomsky Conjecture (Borer 1984).

The paper is organized as follows. In Section 2, I present the data and the empirical boundaries of the present investigation. In Section 3, I go through the main approaches and proposals regarding the analysis of indefinite PAs in Romance languages. Section 4 is dedicated to an overview of nanosyntax, the framework adopted for the present contribution. Section 5 presents the core of the argument, where I offer a novel approach to indefinite PAs and their crosslinguistic variation, especially focusing on their competition with BNs and definites. In Section 6, I highlight a shortcoming of the proposed approach related to the divide between structural and oblique cases, suggesting a possible solution. Finally, Section 7 draws some conclusions.

## 2 Crosslinguistic variation

In this section, I provide an overview of the crosslinguistic variation addressed in this paper, specifying the limits of the current analysis. From a syntactic-semantic point of view, I focus on the expression of two kinds of indefinites. The first type is represented by indefinites referring to an open-ended quantity, whether mass or count, ranging over any possible subset of the referent set denoted by the property-noun. More concretely, I look at the externalization of sentences like *I’ve been [building houses/cooking rice] since I was born*, examining how *houses*/*rice* is lexicalized in different languages. As a second type of indefinites, I focus on those referring to indefinite quantities (numbers or amounts) whose consistency is determined but unspecified (*I baked [some] bread*). I define these two indefinites in a more precise way in Section 5.2, but the general idea is that in the second context we have a boundary defining the indefinite quantity we are dealing with, while in the first case this boundary is absent (see Pinzin and Poletto 2022a, 2022b for other data; Tortora 2008 for an analysis of [un]boundedness in the spatial domain). Henceforth, I refer to the first type as *unbounded indefinites*, while I refer to the second type as *bounded indefinites*. I further constrain the analysis to positive environments (see Baldi and Savoia 2022; Garzonio and Poletto 2020; Savoia and Baldi 2023 for an overview of the negative patterns in the Italo-Romance varieties) and to the Romance varieties in which one of or both the highlighted indefinite types present either *DE *+* def* (= indefinite PAs) or the bare preposition *DE*, as defined in the following paragraph.

I use a set of descriptive labels to identify potentially similar objects in different languages, which consistently appear in the relevant indefinite contexts. With the label *DE* I refer to the prepositions derived from Latin *de* ‘of’ used in genitive contexts, as marking the possessor in possessive contexts. I extend this descriptive label to every occurrence of this form, irrespective of other properties.

(6)

**Table:** 

*La*	*casa*	*di*	*Piero.* (It.)
the.f.sg	house	of	Piero
‘Piero’s house.’			

With the label *def* I refer to definite determiners, the set of markers which appear when the DP refers to a maximal entity (see Section 4). Again, I extend this label to every occurrence of these forms, irrespective of other properties.

(7)

**Table:** 

*Ho*	*incontrato*	*la*	*vicina*	*di*	*Ugo.* (It.)
have.1sg	met	the.f.sg	neighbor	of	Ugo
‘I met Ugo’s neighbor.’					

With the label *DE *+* def* I refer to markers consisting of the conflation of a *DE* element with a *def* element (e.g., Fr. *de la* [f.sg], *du* [m.sg]; Emil. *adla* [f.sg], *dal* [m.sg] etc.), as, for example, when the possessor is referentially definite in a possessive relationship.3The *DE *+* def* morphology is not always analyzable as the juxtaposition of the language specific form for *DE* and for *def*. A clear case is French when it comes to masculine singular derivations in which the following noun starts with a consonant. In these cases, the final form is not the juxtaposition of the expected /də/ and /lə/, but /dy/. The processes deriving /dy/ apply irrespective of *DE *+* def* introducing an indefinite (as in [2]–[3]), a genitive PP that contains a definite (as in [4]) or a partitive (as in [5]).

(8)

**Table:** 

*La*	*casa*	*della*	*vicina.* (It.)
the.f.sg	house	of.the.f.sg	neighbor
‘The house of the neighbor.’			

Finally, with the label bare noun (BN) I refer to the cases in which the noun is not introduced by any overt marker, including *DE*, *def*, or their combination.4Note that I will not consider adjectives in this contribution (but see footnote 36), since our dialectal data on indefinites cover very few cases in which we find the noun modified by an adjective. Considering this, I reserve analyzing the influence that adjectives may have on such configurations in these varieties to further studies. For a diachronic analysis considering these cases in French, see Carlier and Lamiroy (2014), where it is shown that prenominal modifiers disfavor the presence of PAs.

(9)

**Table:** 

*Mangio*	*fragole*	*da*	*una*	*vita.* (It.)
eat.1sg	strawberries	from	a	life
‘I’ve been eating strawberries since forever.’				

Romance languages vary with respect to the way in which they externalize bounded and unbounded indefinites by means of *DE*, *def*, and BN. Let us first look at unbounded indefinites (*I’ve been building houses for years*). A first group of languages – exemplified in (10) by Italian – shows BNs in these cases.5There is regional variation in this respect (see Cardinaletti and Giusti 2020). The judgments have been, however, collected from speakers with different regional/dialectal background and allow for glossing over regional variation for the present study.

(10)

**Table:** 

*Costruisco*	*case*	*da*	*anni.* (It.)
build.1sg	houses	from	years
‘I have been building houses for years.’			

This does not hold for every Romance language. A second group of languages, among which French and Emilian, shows *DE *+* def*, see (11)–(12).6Emilian refers here and henceforth to the variety of Dosolo/Viadana represented in the DiFuPaRo database (https://difuparo.linguistik.uzh.ch/). It is a Gallo-Italic variety spoken at the border between Emilia and Lombardy. For more details see the ABOUT/GUIDE section of the database.

(11)

**Table:** 

*Je*	*construis*	*des*	*maisons*	*depuis*	*30*	*ans.* (Fr.)
sbj.1sg	build.1sg	of.the.pl	houses	from	30	years
‘I’ve been building houses for 30 years.’

(12)

**Table:** 

*I*	*è*	*an*	*ke*	*kostruisi*	*adli*	*kà,*	*ma*	*a*	*n*	*ù*
sbj.3pl	is	years	that	build.1sg	of.the.f.sg	houses,	but	sbj.1sg	not	have.1sg
*mai*	*vist*	*atse*	*bröti.* (Emil., Dosolo)						
never	seen	so	ugly							
‘I’ve been building houses for years, but I’ve never seen such ugly ones.’										
(DiFuPaRo 405)										

Finally, there are varieties where we only find *DE*. An example is the Franco-Provençal variety of Stnic, see *də mɛnta* ‘of mint’ in (13).7On Franco-Provençal PAs and their distribution see Ihsane (2022), Kristol (2014, 2016, Stark and Davatz (2022), Stark and Gerards (2020).

(13)

**Table:** 

*De*	*fɔʀijẽ*	*n*	*æn*	*kwiʎɛ*	*də*	*mɛnta*	*œ pœ*	*ˈĩ*	*sən′aː.* (Fr.Pr., Stnic)
of	spring	sbj.1pl	have.1pl	collected	of	mint	for	one	week
‘In spring, we collected mint for a week.’									
(DiFuPaRo 261)									

If we consider bounded indefinites (*I baked [some] bread*) we have a different distribution. In this case, Italian behaves like French and Emilian, in that they all present *DE *+* def*, see (14)–(16):

(14)

**Table:** 

*Ho*	*cotto*	*del*	*pane.* (It.)
have.1sg	cooked	of.the.m.sg	bread
‘I baked some bread.’

(15)

**Table:** 

*J’*	*ai*	*cuit*	*du*	*pain.* (Fr.)
sbj.1sg	have.1sg	cooked	of.the.m.sg	bread
‘I baked some bread.’

(16)

**Table:** 

*U*	*kot*	*dal*	*pan.* (Emil., Dosolo)
have.1sg	cooked	of.the.m.sg	bread
‘I baked some bread.’			
(DiFuPaRo 438)			

The Franco-Provençal variety of Stnic still behaves differently, presenting only *DE* in these environments too, see *də tsu/kar′ɔt* ‘of cabbage/carrot’ in (17).

(17)

**Table:** 

*Dze*	*dʒəndɔ*	*də*	*tsu*	*e*	*də*	*kar′ɔt.* (Fr.Pr., Stnic)
there	add	of	cabbage	and	of	carrot
‘I add some cabbage and carrots.’						
(DiFuPaRo 47)						

All in all, we can see two main patterns for unbounded and bounded indefinites: Italian overtly differentiates between them; French, Emilian, and Franco-Provençal do not. Additionally, Franco-Provençal differs from French and Emilian in that it only shows *DE*, with no *def*. This is represented in Table 1.

**Table 1:** Lexicalization of unbounded and bounded indefinites in the Romance languages under analysis.

	Unbounded indefinites	Bounded indefinites
Italian	BN	DE + def
French, Emilian (Dosolo)	DE + def	DE + def
Franco-Provençal (Stnic)	DE	DE

This typology exhausts the possibilities when looking at the Romance languages that present either *DE *+* def* or *DE* in either bounded or unbounded indefinites.

## 3 How many lexical items do we need?

The literature on indefinite PAs revolves around two main issues: (i) how to account for the crosslinguistic variation we observe in their distribution and (ii) how to account for the co-occurrence of what looks like a genitive preposition (*DE*) and what looks like a definite determiner (*def*) in these indefinite markers. The second issue can be further elaborated in the following way: how many LIs do we need? Can we unify the presence of *DE* and *def* in indefinite PAs with their tokens as used in other contexts? Cardinaletti and Giusti (2016, 2018, Ihsane (2008), Stark (2016), and Storto (2003) provide a negative answer to the question. In short, they propose that we are dealing with complex indefinite markers that do not actually contain any LI connected to the definite article or the genitive preposition. Storto (2003) proposes an analysis of indefinite PAs in terms of “lexical indefinite determiners” (Storto 2003: 330). In the same vein, for Stark (2016) and Ihsane (2008) *DE* in indefinite PAs is the lexicalization of a low classifying head within the functional sequence of the indefinite DP (*ind[ividuation]°* in Stark 2016; *de°* in Ihsane 2008), while *def* is a higher (agreement) marker into which *DE* incorporates. Finally, adopting the same general idea, Cardinaletti and Giusti (2016, 2018 argue that *DE* and *def* are lexicalizations of an indefinite DP structure, with *def* – an agreement marker – in the D° and *DE* in its specifier. Espinal and Cyrino (2021, 2022 differentiate themselves from these analyses in that they assume that there is no distinction between the instance of *def* in indefinite PAs and *def* as generally used for referring to definite entities. In other words, they do without assuming the presence of two different but homophonous *def* LIs for the definite article. In their analysis, indefiniteness is achieved by means of the *DE* element, defined as an abstract operator shifting definites into property-type expressions. To put it differently, they propose that (these kinds of) indefinites are derived from definites by canceling the definiteness restriction via the *DE* operator. While they unify all instances of *def*, they still maintain that *DE* in indefinite PAs is not synchronically related to the other instances of *DE* (genitive PPs/partitives), thereby assuming two homophonous *DE* LIs. In favor of these approaches assuming different LIs there is a set of syntactic arguments showing how indefinite PAs and genitive PPs/partitives have different behaviors and distributions. The first observation, already presented before, is that indefinite PAs have the distribution of direct arguments (subject or object) and not of PPs. A second related fact regards PP extraction (Espinal and Cyrino 2022; Ihsane 2008, 2013; Milner 1978).8Other facts have been shown to support the idea that these instances have a different syntax, such as the use of resumptive pronouns. Specifically, in Italian, dislocated indefinite PAs are resumed by object pronouns, while genitive PPs and partitives are resumed by a different pronoun, *ne*.

(i)

**Table:** 

*Dei*	*ragazzi*	***li***	*ho*	*visti,*	*degli*	*altri*	*no.* (It.)
of.the.m.pl	boys	obj.3pl	have	seen	of.the.m.pl	others	no
‘I have seen some boys, while some others I didn’t.’

(ii)

**Table:** 

*Di*	*quei*	*ragazzi,*	***ne***	*parlo*	*spesso.* (It.)
of	those	boys	ptv	speak	often
‘I often speak about those boys.’

Note, however, that there is crosslinguistic variation in this respect (e.g., in French indefinite PAs are resumed by the same clitic pronoun as the others, *en*, parallel to Italian *ne*, glossed as ptv for ‘partitive’). This makes these arguments less straightforward to handle. More specifically, indefinite PAs do not block PP extraction (18), while partitives and PPs block it (19).

(18)

**Table:** 

*C’*	*est*	*de*	*nos*	*poules*	*que*	*nous*	*mangeons*	*souvent*	*des*	*oeufs.* (Fr.)
it	is	of	our	chickens	that	sbj.1pl	eat.1pl	often	of.the.pl	eggs
‘It is of our chickens that we often eat eggs.’										
(Carlier 2007: 20)

(19)

**Table:** 

*C’	*est*	*de*	*Zola*	*que*	*j’*	*ai*	*lu*	*deux*	*des*	*livres.* (Fr.)
it	is	of	Zola	that	sbj.1sg	have.1sg	read	two	of.the.pl	books
(Milner 1978: 71 ff.)										

While the idea of separate homophonous LIs takes care of these cases in a straightforward fashion, it requires proposing a large-scale homophony “spreading” to different languages, even beyond the Romance continuum.9Estonian and Russian, among others, show the presence of genitive/partitive markers introducing direct object indefinites (see Luraghi and Huumo 2014 for further references). This has been supported in terms of general shared diachronic processes. Carlier (2007) and Carlier and Lamiroy (2014) elaborate a proposal regarding the grammaticalization path of indefinite PAs in French, while Schurr (2021) proposes a comparative diachronic analysis within the Romance continuum (see Seržant 2022 for a wider comparative analysis beyond Romance languages).

In contrast to these approaches, a second set of proposals takes the different instances of *DE* – and not only *def* (Espinal and Cyrino 2021, 2022) – to be synchronically linked to a single LI in the lexicon, albeit with different degrees of “unity”.10Since I only deal with positive environments in this context, I do not consider here Garzonio and Poletto (2020), who differentiate between *DE* under negative and positive polarity and connect only the *DE* appearing in negative contexts to the genitive lexical item. Some authors focus only on partitives versus indefinite PAs, unifying them and leaving aside the occurrences of *DE* as a genitive preposition. Chierchia (1997) and Zamparelli (2008), for example, propose an analysis of Italian indefinite PAs that implies this kind of partial unity. The gist of these proposals is that *DE* performs the same function in both partitives and indefinite PAs. More specifically, for Chierchia *DE* performs no semantic function, so that it has no meaning in either case, while in both cases *def* performs the same function it generally does in the language. This in turn means that, in his approach, there is no semantic difference between partitives and indefinite PAs. On the other hand, for Zamparelli, *DE* lexicalizes the same semantic operator, labeled R for “residue”. This operator returns the denotation of its specifier minus the denotation of the complement. The difference between partitives and indefinite PAs is then related to the denotation of the complement subject to the R operation: as a complement of R, partitives have a “regular” definite referring expression, while indefinite PAs have a kind-denoting DP (introduced by definite articles in this language; see footnote 1). Both Zamparelli and Chierchia do not explicitly account for the syntactic differences between indefinite PAs and partitives shown in (18)–(19) and for the occurrence of *DE* in genitive PPs (see [4], [6]–[8]).11See Storto (2003: 323–328) for an in-depth analysis of various issues related to Chierchia’s proposal. In addition, Zamparelli’s proposal does not directly account for the interpretational difference between indefinite BNs and *DE *+* def* in Italian: if the R (“residue”) operation applies vacuously, there should be no difference between the denotation of the specifier (indefinite BN) and the denotation of the whole structure (indefinite *DE *+* def*). This is not correct, as the distribution of these nominal phrases in Italian shows (see Pinzin and Poletto 2022a, 2022b).
Manzini (2019), finally, proposes a generalized unity hypothesis. The core idea is that the LI for *DE* is always an element carrying relator content, defined as imposing a part/whole relationship (⊆). Its syntactic label is that of a preposition (P). When this element merges with a complement DP (e.g., [_P_ DE] + [_DP_ la viande]), the newly created constituent {DE, DP} is either labeled as a PP following *DE* ([_PP_ [_P_DE] [_DP_ la viande]]) or as a DP following *la viande* ([_DP_ [_P_ DE] [_DP_ la viande]]). The choice between the two is context-dependent. Adopting this approach, the syntactic problem related to the extraction facts highlighted in (18)–(19) disappears. If the *DE* part/whole relator can be merged with a complement DP and the new syntactic object can receive the same label as the complement DP itself, then it can be selected by a verb, both in object and subject position. Along the same lines, it could be claimed that labeling the whole constituent PP blocks the extraction of a PP merged within it.12Note that Manzini (2019) follows the same semantic analysis presented by Zamparelli (2008): the part/whole relator *DE* relates a complement DP_Kind_ with the plural/mass property in [spec,DP], providing the count/mass property itself as output.

In short, we see a continuum of proposals ranging from the ones advocating for the necessity of having different homophonous LIs for both *def* and *DE* in the lexicon (Cardinaletti and Giusti 2016, 2018; Ihsane 2008; Stark 2016; Storto 2003; plus the diachronic analyses in Carlier 2007; Carlier and Lamiroy 2014; Schurr 2021) to the proposals assuming that both the *def* and the *DE* we find in indefinite PAs are the same elements we generally see in the language in other contexts (Manzini 2019). As a middle ground, Espinal and Cyrino (2021, 2022, Chierchia (1997), and Zamparelli (2008) support different degrees of “unification”.

The crosslinguistic variation within Romance languages is only modeled by a subset of these proposals: Cardinaletti and Giusti (2016, 2018, Espinal and Cyrino (2021, 2022, Stark (2016).13Pinzin and Poletto (2022a, 2022b), Schurr (2021), and Stark (2008a, 2008b) present data on the crosslinguistic variation but do not propose a formal synchronic model that accounts for the variation. While Stark (2016) draws a connection between the expression of plurality on the noun and the distribution of BNs and PAs in Romance languages (see also Delfitto and Schroter 1991), the other proposals relate the crosslinguistic variation to different language-specific lexicalization choices of the same structure. Crucially, no account assuming partial or total unity for the occurrences of *DE* (Chierchia 1997; Manzini 2019; Zamparelli 2008) addresses the issue of the crosslinguistic variation observed in Section 2. In what follows, I put forward a proposal which unifies the occurrence of *DE* and *def* in both unbounded and bounded indefinites with their other occurrences under a single LI for each, while at the same time accounting for the observed crosslinguistic variation.

Before going into the analysis, a few remarks on the adopted framework are in order.

## 4 Nanosyntax, an overview

Nanosyntax (Starke 2009, 2014) is a realizational model for the lexicon-syntax interface based on constituency that assumes that syntax is the only combinatorial mechanism within the set of modules producing linguistic expressions.14This means that no pre- or post-syntactic combinatorial mechanism deriving complex words or feature bundles is assumed to be present. The label “realizational” is shared with other approaches such as distributed morphology (Halle and Marantz 1993) and means that LIs are seen as *realizing* (= lexicalizing) syntactic-semantic units, which are merged previously and independently of them.15The opposite view is often called representational because LIs are seen as the basis for the syntactic computation, therefore directly *representing* the syntactic-semantic features. In addition, nanosyntax differs from distributed morphology in having no feature bundles in the pre-syntactic lexicon (only atomic syntactic-semantic features) and no post-syntactic morphological operations (merge/fusion/fission etc.), see Caha (2018) for an overview of the main differences. Syntactic-semantic units are conceived as a set of content-bearing features (see [20]) combined by the speaker to output complex meanings.16No specific claim is made about the language-specific nature of these units of meaning, which could well be shared with other cognitive processes. Language is a way of externalizing these units and their meaningful combinations. The combinatorial mechanism is assumed to be restricted to binary merge. This merge operation, being binary, operates by taking a single feature and merging it either with another feature or with the result of a previous merge operation. The result of a series of merging operations is therefore a nested set of features (21), referred to in syntactic terms as *constituent* or *phrase*.

(20)

**Table:** 

{x, y, z …}

(21)

**Table:** 

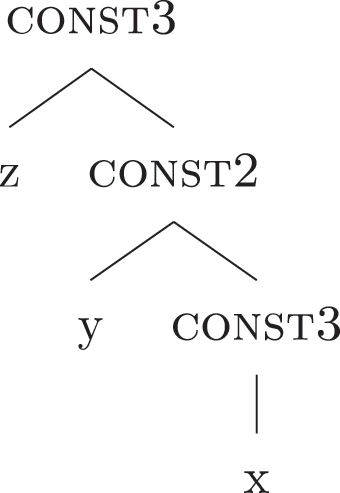

The order of merge of the different syntactic-semantic features is guided by the final meaning the speaker wants to express. Each merge operation modifies the meaning of the constituent built by the previous merge operation. A set of constituents in a specific order (and, therefore, with a specific meaning) makes up a *functional sequence* (*fseq*). For example, Caha (2009, 2013 advocates for a specific *fseq* for case systems, in which different cases correspond to different constituents in a containment relationship.17The hierarchy is based on a crosslinguistic survey of case syncretisms (see sources in Caha 2009, 2013). The survey shows that there is a restriction on the attested syncretisms, which is captured by proposing the universal *fseq* adopted in the present analysis. More concretely, the attested syncretisms only target contiguous pieces of the *fseq*, so that, for example, no nom/gen syncretism is attested to the exclusion of acc. What we generally label *nominative* corresponds to a constituent containing the feature {f1} ([nomP]), *accusative* corresponds to a constituent containing [nomP] and {f2} ([accP]), *genitive* corresponds to a constituent containing [accP] and {f3} ([genP]) and so on.18In the rest of the contribution, I will use curly brackets to single out features {f} and square brackets for phrases [fP].

(22)

**Table:** 

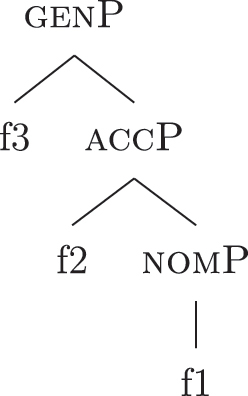

This means that a constituent containing only {f3} will not be interpreted as genitive. It is the composition of three features that is interpreted as such. In this case, the different *fseqs* for each case are different from one another because of the presence/absence of a feature *on the top* of the derivation. However, when we enlarge the picture to larger segments of the structure, it is easy to realize that *fseqs* frequently diverge because of a feature *in the middle* and not because of a feature on the top. In other words, different *fseqs* are generally opposed to each other because of *gaps*: one of the two *fseqs* has an additional feature in the middle of the sequence with respect to the other. Such gapped structure becomes visible when one adopts a layered structure for the (in)definiteness features, in line with Ihsane (2008, 2013 and Zamparelli (1998, 2000, among others. To see why, let us further propose that a definite derivation builds on an indefinite one by adding a feature (for a similar analysis see Zamparelli 2000). This additional feature can be thought of as imposing a maximality restriction {max} on the denotation of the noun (Fintel et al. 2014; Link 1983; Sharvy 1980; Wągiel 2021; Zamparelli 1998, 2000; see Section 5 for more details).19But see Espinal and Cyrino (2021, 2022 for a different proposal, in which definites are the basis for deriving indefinites by means of an abstract type-shifting operator.

(23)

**Table:** 

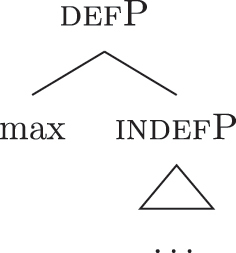

As commonly and independently assumed (see Lamontagne and Travis 1987; Picallo 1991, among others), the case *fseq* closes-off the nominal domain and is merged on top of the (in)definiteness one. In this situation, a “definite nominative” *fseq* – in (24) – will be different from an “indefinite nominative” one – in (25) – because of an additional feature in the middle.

(24)

**Table:** 

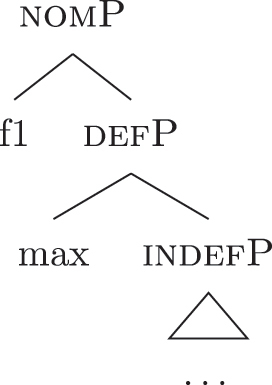

(25)

**Table:** 

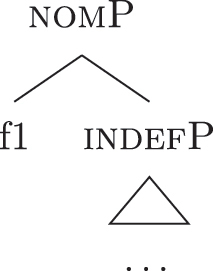

Note that the label of the top constituent (“nomP”) is the same in both cases. This is a shortcut, adopted for expository reasons: the two “nomPs” constituents are different: one contains {max}, while the other does not.20A final logical possibility is that two *fseqs* diverge because of two incompatible features, as in the following scheme, where one *fseq* contains {a} and the other contains {b}.

**Table:** 

i.	[a [y [x]]]
ii.	[b [y [x]]]

As the data will not lead me to adopt such a hypothesis for any derivation, I leave this possibility aside.

As far as syntax *per se* is concerned, without taking into consideration the relationship with the lexicon, nothing more needs to be added: the syntactic component takes as input content-bearing syntactic-semantic features and gives as output nested sets of these features derived by a binary merge operation. In standard nanosyntactic practice, no crosslinguistic variation is attributed to this part of the linguistic computation. Languages are maximally homogeneous with respect to the available set of syntactic-semantic features and their ordering, so that a linguistic expression that has the same interpretation in two different languages will contain the same set of features in the same order, i.e., *fseqs* are universal (in line with cartographic approaches, see Cinque 1999; Rizzi 1997). All the crosslinguistic variation should be attributed to the language-specific lexicon involved in the externalization procedure, still to be outlined.21Note that the nanosyntactic mechanisms to be described in the following paragraphs (constituent lexicalization + lexicalization algorithm) do not logically imply such an approach to crosslinguistic variation. One could assume that (i) languages might vary with respect to the set of available syntactic-semantic features and/or (ii) two partially different *fseqs* (i.e., different features, different orders, etc.) might end up having the same semantic interpretation and still adopt the same mechanisms.

The externalization procedure takes care of lexicalizing the nested sets of syntactic-semantic features derived by syntax by putting them into communication with the lexicon of the language, i.e., a list of LIs. LIs are conceived as tripartite entities (26a) containing an externalization form (for spoken languages this is generally equal to a phonological representation), syntactic-semantic information and conceptual content, holding the information that is not encoded by syntactic-semantic features.22Not all LIs contain a content (e.g., the English LI /z/ for plural). The syntactic-semantic information is stored in the same form in which they appear in syntax: nested sets of features, i.e., constituents (“the (morpho)syntactic lexicon contains nothing but well-formed (morpho)syntactic expressions” [Starke 2014: 1]).23Constituents are labeled following the last feature merged, despite possible differences regarding lower portions of the *fseq* (see [24]–[25], both labeled nomP in line with the last feature merged despite differences between their *fseqs*). Since the conceptual content is not relevant in the following discussion, I simplify LIs as in (26b) and refer to them by means of the externalization form, such as *a* in (26b).

(26)

**Table:** 

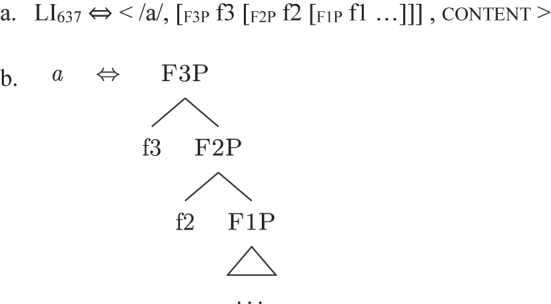

The lexicalization (= externalization) procedure is cyclic and is triggered every time a feature is merged with the *fseq* at a given point in the derivation. Each time a feature is merged, the lexicalization procedure searches the lexicon for a LI matching the constituent derived by the syntactic computation. Each feature must be lexicalized for the derivation to be successful. A LI is considered an adequate match for a given constituent if it *contains* that constituent, but it can contain other features as well. This is the reasoning behind what is generally known as the Superset Principle in nanosyntax: “A lexically stored tree matches a syntactic node iff the lexically stored tree contains the syntactic node” (Starke 2009: 3). Let us pretend our toy language has the LI *a* in (26b) in its lexicon and that syntax derives [f1P], as in (27). In this situation, the LI *a* matches the syntactic derivation and lexicalizes it, as highlighted.24The syntactically derived constituent is represented on the left, the LIs on the right. If one or more LIs match the syntactically derived constituent, the matching portion of the LI(s) is surrounded by a square. The phonological form of the winning LI lexicalizing the constituent is shown under the syntactic constituent, surrounded by a circle.

(27)

**Table:** 

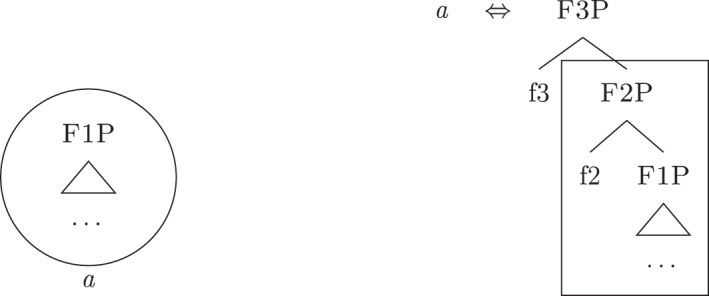

The syntactic component can then derive another constituent by merging {f2}, as in (28). The same LI *a* containing {f1} provides a suitable option in this case too, so that the lexicalization procedure is satisfied.

(28)

**Table:** 

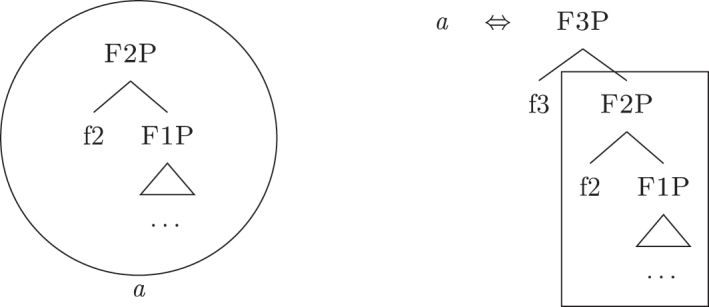

By these principles, the single LI *a* can lexicalize more than one *fseq*, giving rise to non-accidental syncretism or coexpression (i.e., more functions, one entry).

For now, we have only seen cases in which a single LI is adequate for lexicalization. It could be the case, however, that more LIs match the constituent derived by syntax. Let us enrich our lexicon with the LI *b*, as in (29). Given the same syntax as in (28), both *a* and *b* are adequate lexicalizations; they “compete”. The resolution of this competition comes from the application of the Elsewhere Principle (Kiparsky 1973, among others): use the more specific LI. Since *b* applies to a subset of the contexts in which *a* applies, *b* is more specific and wins the competition.

(29)

**Table:** 

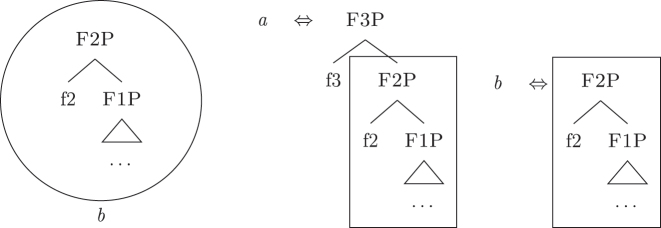

This principle is relabeled in nanosyntactic terms as Minimize junk (“At each cycle, if several LIs match the root node, the candidate with least unused nodes wins” [Starke 2009: 4]).25Note that if we then merge {f3}, the only available candidate will be *a*, since it would be the only LI matching the constituent derived by syntax.

Another possibility is that no LI matches the constituent derived by syntax as it is. Here one of the core principles of nanosyntax comes into play: the cyclic lexicalization procedure *must* find an adequate match and each feature must receive an adequate lexicalization at each cycle, otherwise syntax does not proceed merging another one. To achieve this goal, the lexicalization procedure goes through a series of steps (i.e., an algorithm) to “fix” the syntactic derivation. After each step the lexicon is searched to see if it was successful. As soon as a step is successful (i.e., all features receive a lexicalization), the procedure is satisfied, and the syntax can go on merging another feature. The algorithm is assumed to be universal, so that no crosslinguistic difference is supposed to be encoded here. In this contribution I adopt the following version of the algorithm, argued for in Caha (2019) (but see Starke 2018).

(30)

**Table:** 

Merge {f}:		
a.	Lexicalize [fP].	
b.	Lexicalization-driven movements:		
	i.	Spec-to-spec: Move the Spec of the complement of {f}. Retry (a).
	ii.	Complement-to-spec: Move the complement of {f}. Retry (a).
c.	Backtracking:	
	i.	Go back one step in the derivation and try the next option for that cycle.
	ii.	Try to merge {f} in the complex left branch and lexicalize it.
d.	Complex left branch: spawn a new subderivation providing {f}, lexicalize it and merge it with the main derivation, projecting [fP].		

Up until now we have only seen lexicalizations successful at step (a), so that the following steps of the algorithm were not triggered (see [27]–[29]). The steps in (b) involve lexicalization-driven movements, i.e., the movement of either the Spec (b.i) or the complement (b.ii) of the syntactically derived constituent. These “rescue” movements change the geometry of the tree and give lexicalization another opportunity to succeed. The step in (c.i) involves going back one lexicalization cycle and choosing the next-best step available, a way to undo a derivation that led to a dead-end. For a discussion of these mechanisms and a potential alternative to backtracking see Blix (2021). I will not discuss them in any more detail here since steps (a.)–(c.i) will not be relevant in the discussion. This is because no step until (c.ii) will ever be successful with the kind of LIs involved in the lexicalization of the nominal phrases described in Section 2 (*DE*, *def*, etc.). On a descriptive level, these LIs always linearly precede the lexical core of the *fseq* – the *noun* LI – so that they can be defined, following Starke (2018), as “pre” LIs or *complex left branches*. In the current understanding, the distinction between the LIs preceding (“pre”) and following (“post”) the lexical core of the *fseq* is driven by the complexity of the structure below the lowest non terminal node (*foot* henceforth) of the constituent with which they are stored in the lexicon: “post” markers have a unary foot (see [31a], where [f1P] dominates only {f1}), while “pre” markers/complex left branches have a binary foot (see [31b], where [f1P] dominates {f1} and x26The status of x is tangential to the present argumentation and deliberately left vague. I will use it for all complex left branches I will present. It could be a feature of the main *fseq* on the right or an independent feature which functions as an unmarked predicative base. I leave this question open for the time being.).

(31)

**Table:** 

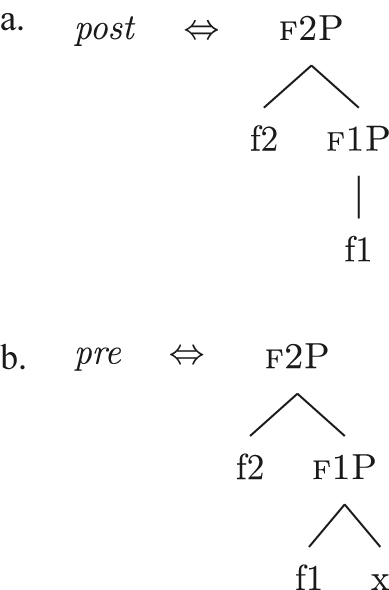

This follows from the mechanics of lexicalization-driven movements. If any kind of lexicalization-driven movement displacing parts of the *fseq* to its left has happened, the right constituent will necessarily have a unary foot. This is because lexicalization-driven movements vacate the position from which they start without leaving an interpretable trace.27These movements are of the “meaningless” kind proposed by Cinque (2005). They differ from “feature-driven” movements, as Wh/Focus-movements, which have a semantic import. On the other hand, this means that a LI with a binary foot as (31b) will never match any right constituent derived by lexicalization-driven movements. Since the LIs currently composing our lexicon are only ever used as “pre” markers and therefore have a binary foot, steps (a)–(c.i) of the algorithm will never be successful. This allows us to skip these steps for the rest of the contribution.28Note that given that we are dealing with an algorithm, they will be attempted and fail each time a feature is merged: they will never be skipped. As for the two remaining steps, the idea is that in case a complex left branch is already present in the derivation, the algorithm will first try to expand it with the new feature (c.ii), then, if this does not lead to a successful lexicalization, a new complex left branch will be derived in an auxiliary workspace (d). In case a complex left branch is not already present in the derivation, step (c.ii) vacuously applies and a new complex left branch is derived in an auxiliary workspace (d). In the next sections I will adopt these mechanisms to derive the patterns observed in Section 2, starting with unbounded indefinites (see Table 1).

## 5 Genitive and definite markers lexicalizing indefinites

Let us recall the main issues posed by indefinite PAs. We have what appears to be a genitive marker (*DE*) appearing in a context in which we would expect no genitive feature (nominative/accusative arguments) and what appears to be a definite article (*def*) in a context in which we would expect no definiteness (indefinite nominal phrases). This is illustrated with Emilian *adli kà* ‘houses’ in (12) repeated below as (32).

(32)

**Table:** 

*I*	*è*	*an*	*ke*	*kostruisi*	*adli*	*kà,*	*ma*	*a*	*n*	*ù*
sbj.3pl	is	years	that	build.1sg	of.the.f.sg	houses,	but	sbj.1sg	not	have.1sg
*mai*	*vist*	*atse*	*bröti.* (Emil., Dosolo)							
never	seen	so	ugly							
‘I’ve been building houses for years, but I’ve never seen such ugly ones.’										
(DiFuPaRo 405)										

In line with part of the literature (Carlier 2007; Ihsane 2008; Stark 2016), I argue that the structure in (32) does not contain genitive and definite features interpreted as direct structural case and indefinite, respectively. On the contrary, I contend that *DE* and *def* in (32) lexicalize neither genitive nor definiteness features. I follow the analysis of Caha (2009, 2013 for case features, in which the different cases correspond to different cumulative sets of features (see Section 4, example [22]). I adopt a similar containment hypothesis for the features regulating (in)definiteness: definite reference is built on the syntactic structure that yields indefinite reference by restricting the possible interpretations to a strictly maximal object in the context (Fintel et al. 2014; Link 1983; Sharvy 1980; Wągiel 2021; Zamparelli 1998, 2000).29The maximality approach has been developed to account in a unified manner for the definiteness restriction with nouns whose extension contains count (singular and plural) and/or mass objects. Implications of uniqueness are treated as a side effect. A maximal object for predicate x is defined as such if there is no object x ordered higher than it. Different ordering criteria have been considered in the literature. In Sharvy’s (1980) original proposal, the ordering criterion is based on the part/whole relationship between the objects for which the noun predicate is true. In Fintel et al. (2014), the ordering criterion is informativity (i.e., the object creating the most informative proposition is maximal). In this section I only deal with what I defined before as unbounded indefinites, that is indefinites of the kind found in sentences like *I’ve been building houses/eating rice for years*. The feature building the denotation of unbounded indefinites is labeled {indef}, which will serve as a basis for the derivation of bounded indefinites too (see Section 5.2). Below it, we have the predicative core of the nominal structure, labeled NP.30NP is a convenient shorthand for the whole portion of the structure where (direct) nominal modification also takes place. The difference between the denotations of NP and indefP follows the intuition in Dobrovie-Sorin and Beyssade (2012), where it is claimed that mass and plural BNs, at least in Romance, are amount-referring expressions containing an existential quantifier (see Longobardi 2001 for a similar approach). This accounts for the fact that they can occur as arguments, in contrast to pure property-denoting expressions like adjectives. In (33a) we have an accusative indefinite *fseq* and in (33b) an accusative definite *fseq*.

(33)

**Table:** 

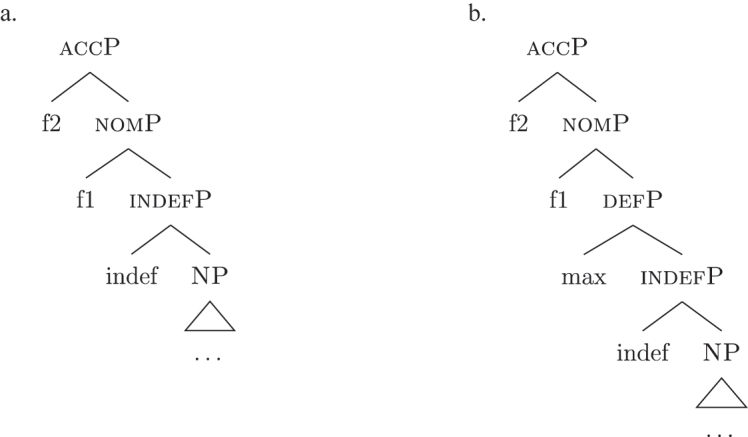

The appearance of *DE* and *def* LIs as a lexicalization of (33a) is due to the fact that these LIs lexicalize complex constituents which not only contain the features for genitive/definiteness, but also the lower features for nominative/accusative ({f1}–{f2}) and indefiniteness ({indef}). By the Superset Principle, these LIs can lexicalize these lower features too. In (34) I present a first approximation of the LIs *ad* (*DE*), *al* (*def*), and the *noun* in (Mantovano) Emilian.31I use Mantovano Emilian here because the compositionality between *DE* and *def* is transparent in this language, for all gender/number combinations. The form of the definite determiner is the following: m.sg /al/, f.sg /la/, m.pl /i/, f.pl /li/, while the form of the *DE* is /ad/. The form of *DE *+* def* is: /dal/, /adla/, /di/, /adli/. The vowel /a/ of /ad/ disappears when the definite determiner begins with a vowel. In other languages, like French, this is not the case (see footnote 3). Note that *ad* and *al* have a binary foot and therefore can only be used as complex left branches. I leave gender and number aside and present for *def* a single LI with the externalization form of the masculine singular, *al*.32For an in-depth analysis of number, gender, and class features in Italian from a nanosyntactic perspective see Janků and Starke (2020). The analysis I am presenting in this contribution is in principle compatible with their analysis. However, to derive the whole set of data, such an analysis would need to be paired with a general account for the spreading of these features in the nominal domain (aka *concord*). See Caha (2019: 267–287) for an approach to the mechanism of concord within nanosyntax.

(34)

**Table:** 

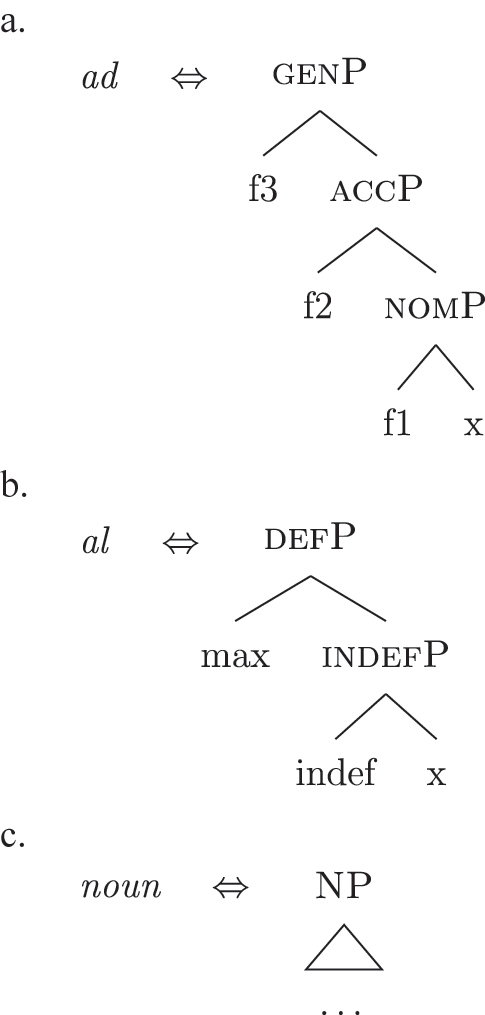

By means of the Superset Principle, *ad* and *al* can lexicalize any of the constituents they contain: *ad* can lexicalize both [accP] and [nomP], while *al* can lexicalize [indefP].33The languages I am dealing with have an “indefinite article” too, the form of which is either identical or related to cardinal *one*. While for reasons of space I am not including it in the analysis, note that its LI is marked for a set of features that is different from the ones proposed for the definite article. Besides not being capable of lexicalizing {max}, it is also constrained to count contexts, while the definite article appears both in mass and count contexts (It. *l’acqua* ‘the water’ and *l’amica* ‘the friend’). Given this different distribution, one could model the LI of the indefinite article as including a quantity/numerosity-restriction (in terms of Dobrovie-Sorin and Beyssade 2012, this kind of expressions would contain an additional feature restricting existential quantification to amounts of cardinality 1). A more precise model is however left open for further studies. Hence it is not surprising to find them introducing nominative/accusative unbounded indefinites. In this case, the question is why we do not see these LIs everywhere. How can we constrain *ad* and *al* to lexicalize the indefinite *fseq* in (33a)? Without further specifications, we would expect syncretism between definite (33a) and indefinite (33b) *fseqs*: each of them should be lexicalized as *ad *+* al *+* noun*. Let us see step-by-step how under the current assumptions (33a) and (33b) would come to be lexicalized by the same LIs. As a first step let us start from the NP – the predicative core – shared by the derivations of definite and indefinite *fseqs* alike. The syntactic component derives the NP and looks for a lexicalization, finding the *noun* LI in the lexicon of the language (for which, see [34]), as shown in (35).

(35)

**Table:** 

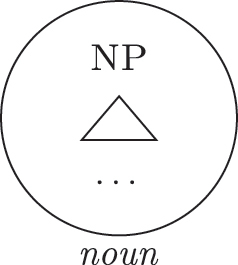

When {indef} is merged, as shown in (36), the lexicon is searched again for an appropriate lexicalization.

(36)

**Table:** 

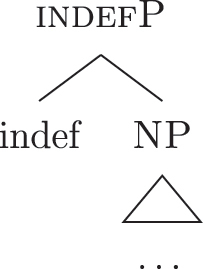

The steps of the lexicalization algorithm in (30) from (a) to (c.i) fail since no LI with a unary foot is present in the lexicon. Step (c.ii) fails because there is no complex left branch already available. The only option is (d) – opening an auxiliary workspace and deriving a constituent closed by the feature to be lexicalized ({indef}) – and (37) is derived. (37) can be fully lexicalized by *al* in (34b) for the left constituent and by *noun* in (34c) for the right constituent.

(37)

**Table:** 

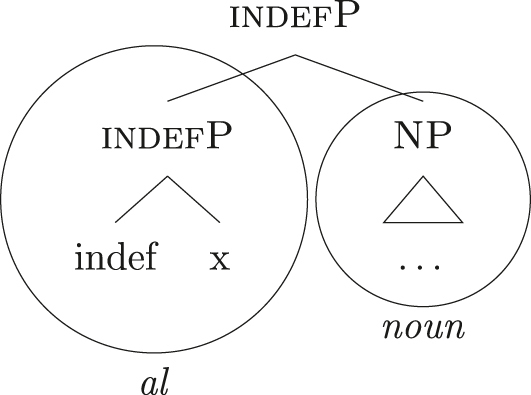

At this point, the definite and indefinite derivations diverge. In the indefinite derivation in (38) case features are directly merged, starting with {f1} deriving [nomP].

(38)

**Table:** 

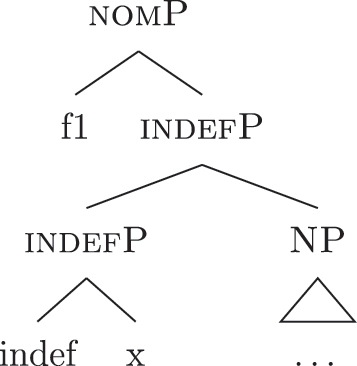

As before, the steps from (a) to (c.i) fail because there is no LI with a unary foot in the lexicon. Step (c.ii) is then attempted and {f1} is merged on top of the complex left branch, as (39) shows.

(39)

**Table:** 

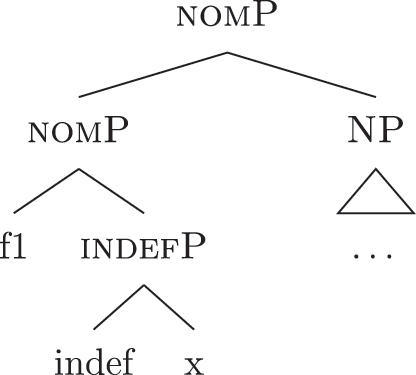

In this case too, however, there is no possible lexicalization: no LI in the lexicon in (34) matches such a complex left branch. Step (d) is then attempted, leading to the creation of another complex left branch with {f1} as its highest feature, as in (40). Given the presence of *ad* in (34a), and assuming the Superset Principle, (40) can be fully lexicalized as that complex left branch is contained within (34a).

(40)

**Table:** 

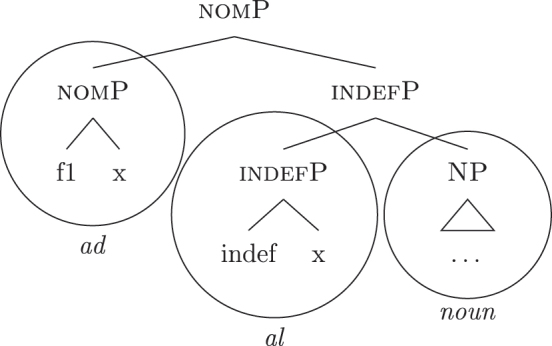

With a successful lexicalization, the next case feature {f2} can be merged and the derivation can proceed (I skip this part of the derivation for reasons of space; in this case, step [c.ii] is successful: the highest complex left branch is expanded with {f2} and *ad* lexicalizes it).

Let us see what happens with a definite derivation, starting from where we left it in (37). In this case, {max} is merged before case features, deriving (41).

(41)

**Table:** 

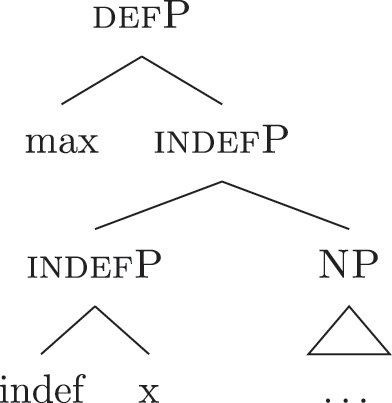

The steps from (a) to (c.i) fail and (c.ii) is attempted, merging {def} on top of the complex left branch. Given the presence of *al* (34b) in the lexicon, the step is successful, yielding (42).

(42)

**Table:** 

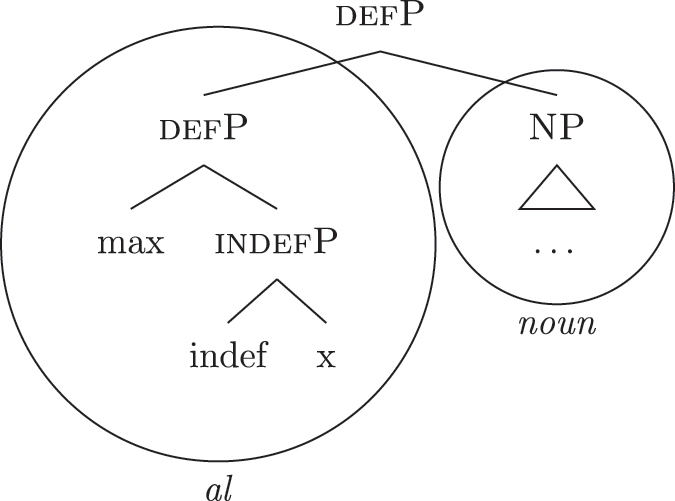

At this point, case features are merged, starting from {f1}. In our lexicon in (34), only *ad* contains {f1}. Nothing should therefore change from (40): only step (d) can be successful, yielding a new complex left branch lexicalized by *ad*, as in (43).

(43)

**Table:** 

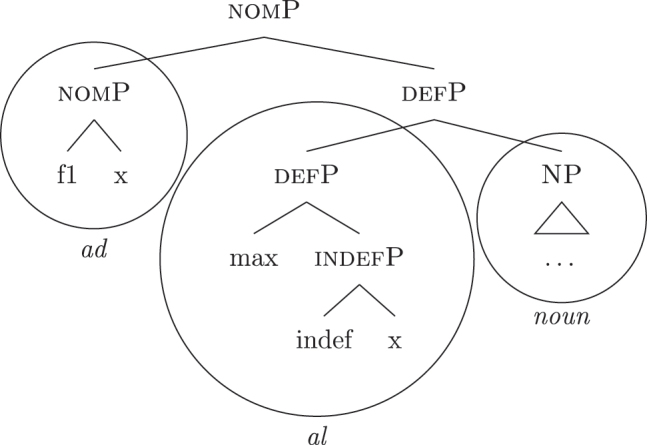

This is not adequate, however, given that in these languages a definite *fseq* is lexicalized as *al* + *noun* and not as *ad* + *al* + *noun*, as shown by *al pütel* ‘the boy’/*li sigoli* ‘the onions’ in (44).

(44)

**Table:** 

*Al*	*pütel*	*a*	*i*	*à*	*butadi*	*via*	*li*	*sigoli.* (Emil., Dosolo)	
the.m.sg	boy	sbj.3sg	obj.3pl	has	thrown	away	the.f.pl	onions
‘The boy threw away the onions.’								
(DiFuPaRo 597)								

The analysis makes incorrect predictions. As a first approach, one could postulate the existence of a phonologically null LI for nominative/accusative case, as in (45). This LI would outcompete *ad* in (34a) for the lexicalization of {f1}–{f2}, being more specific (by Minimize junk).

(45)

**Table:** 

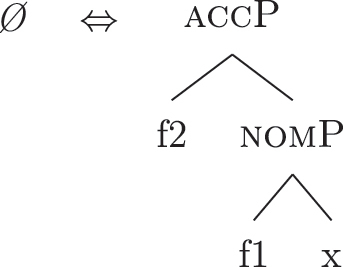

This solution would produce the expected *Ø *+* al *+* noun* lexicalization for the definite *fseq* in (33b), but it would also produce the same *Ø *+* al *+* noun* lexicalization for the indefinite *fseq* in (33a), leading to incorrect predictions again. The relevant fact to consider is that the presence of *ad* depends on the presence in the *fseq* of the feature {max}. That is, *ad* only appears when *al* does not lexicalize {max}, while *ad* is not necessary when *al* lexicalizes {max}. Constituent lexicalization gives us a straightforward explanation for this behavior. Let us propose that the LI *al* can lexicalize, on top of {max}, the nominative/accusative case features {f1}–{f2} too.34As a potential expansion, one could take into consideration languages such as the Apulian Romance variety of San Gargano in Lamis (Massaro 2022), where the definite article can – in specific syntactic contexts – lexicalize the genitive layer too, so that no genitive preposition is present. As an illustration, the following example expresses possession with no overt genitive marker.

(i)

**Table:** 

*Li*	*libbra*	*l-a*	*skɔl-a.* (Apulian, San Gargano in Lamis)
the.pl	book.m.pl	the.f.sg.gen	school.f.sg
‘The schoolbooks.’			
(Massaro 2022: 2)

Calabrese varieties (Rohlfs 1969; Silvestri 2016) have been described as having similar grammatical patterns, albeit partially different, since in these varieties the relevant factor is the definiteness of the nominal, irrespective of the presence of the definite article (e.g., also proper names show the pattern). We then substitute (34b) with (46) in the lexicon of this language.

(46)

**Table:** 

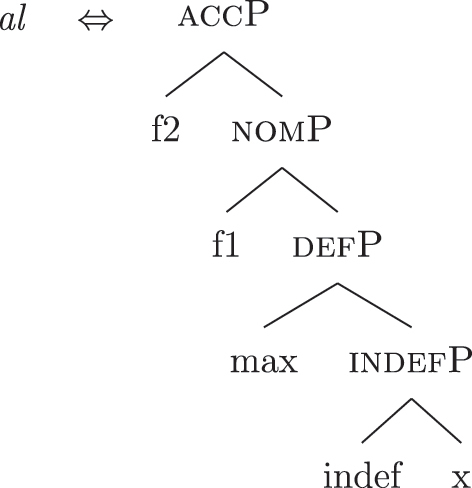

In a definite derivation, where {max} is present, the LI *al* in (46) becomes a competitor for the lexicalization of {f1}–{f2} and outcompetes *ad*. This is because the lexicalization algorithm prefers expanding an already present complex left branch – step (c.ii) – to opening another auxiliary space and deriving a second complex left branch – step (d). The result can be seen in (47), where I show the full derivation up to the accusative feature {f2}.

(47)

**Table:** 

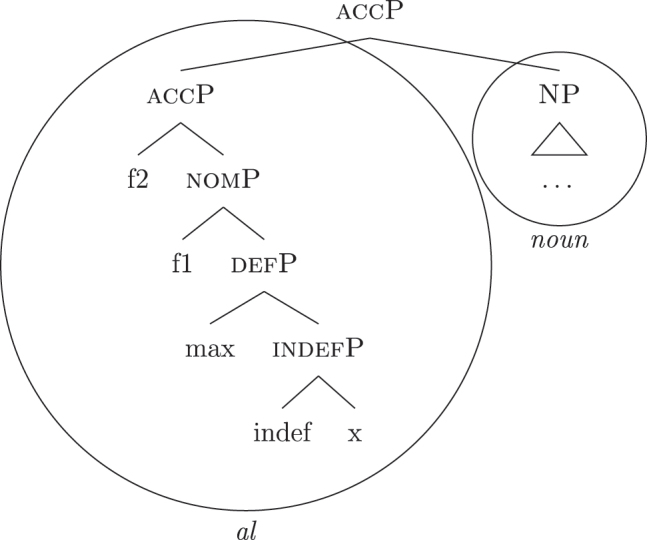

On the contrary, the LI *al* in (46) would not be able to lexicalize {f1}–{f2} in an indefinite derivation. Recall that an LI can only lexicalize a constituent if it contains that constituent. If the derivation lacks {max} and merges {f1} directly with [indefP], the LI containing {max} in its lexically stored *fseq* in (46) is blocked from lexicalizing {f1}. Let us see this in more detail. The indefinite derivation gets to the stage in (38), repeated in (48).

(48)

**Table:** 

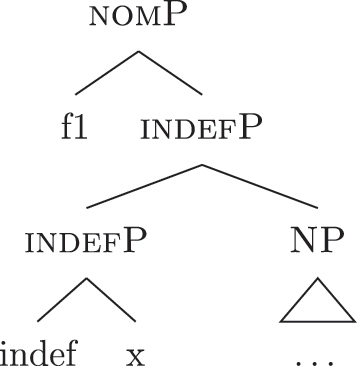

By the logic of the lexicalization algorithm, all the steps are attempted. The steps up to (c.i) fail, as always in the cases at stake given the absence of LIs with a unary foot. Step (c.ii) is then attempted, deriving (49) (repeated from [39]).

(49)

**Table:** 

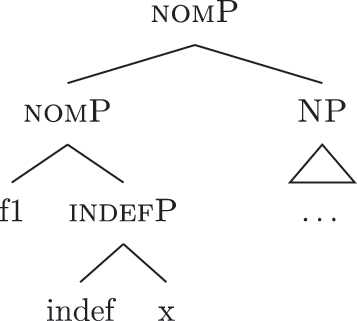

Just as in (39), this step fails. There is no LI in the lexicon containing [nomP] on the left. Even the updated *al* in (46) fails, since [nomP] does not contain {max}, while (46) does. In other words, the gap (absence of {max}) blocks the lexicalization potential of *al* to [indefP], so that *ad* must come into play to lexicalize {f1}, being the only available competitor. The final syntactic configuration predicted for unbounded indefinites in Emilian and French is therefore the one already presented in (40), where the lower layers up to indefP are lexicalized by *def*, while the higher case layers are lexicalized by *DE*.

To summarize thus far, by virtue of two ingredients, (i) the constituent lexicalization algorithm and (ii) the LIs for *def* and *DE* in (46) and (34), we get an analysis for the main issue we started from: indefinite PAs contain definite and genitive LIs without any semantically interpreted definite/genitive feature being present. In doing so, the analysis allows for modeling the different behavior of *DE *+* def* in indefinite PAs versus genitive PPs/partitives observed in (18)–(19): indefinite PAs, unlike genitive PPs/partitives, do not contain any genitive syntactic-semantic feature {f3}. Binding the block on extraction to the presence of non-structural case features like genitive predicts the observed behavior, in line with Kayne (1975: 114–115), who suggests that the block stems from the presence of a PP node (i.e., genitive, in our terms) blocking the extraction of another PP embedded within it. If no PP node is present (i.e., no genitive, in our terms), extraction of an embedded PP is allowed (see also Cardinaletti and Giusti 2016: 65, who reach a similar conclusion assuming that PPs are strong islands for extraction).35Espinal and Cyrino (2022: 189) link this asymmetry to the fact that partitives as in (19) are definites, while PAs are indefinites. In their proposal, the block on extraction would stem from the fact that definite DPs are islands while indefinite DPs are not. Such an approach is compatible with the current proposal too, since indefinite PAs, contrary to partitive structures, contain no definiteness feature {max}.

In the next Section I model the crosslinguistic variation regarding the externalization of unbounded indefinites in the languages of the Italian and Franco-Provençal type (see Table 1).

### 5.1 Unbounded indefinites: crosslinguistic variation

The current analysis accounts for the fact that some languages, such as French and Emilian, lexicalize the *fseq* for unbounded indefinites in (33a) with a *DE* + *def* sequence, without there being any genitive ({f3}) or definiteness ({max}) feature in the syntactic derivation. The final syntactic configuration up to the nominative layer is in (50) (repeated from [40]).

(50)

**Table:** 

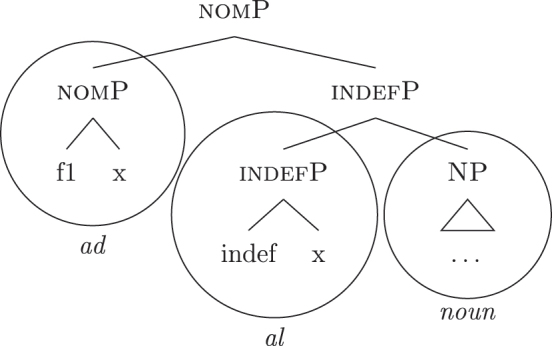

However, in the same unbounded indefinite contexts in which Emilian and French have *DE *+* def*, languages such as Italian have BNs. See (9), repeated here as (51).

(51)

**Table:** 

*Mangio*	*fragole*	*da*	*una*	*vita.* (It.)
eat.1sg	strawberries	from	a	life
‘I’ve been eating strawberries since forever.’				

I propose to relate this crosslinguistic variation to the amount of structure that the nominal root can lexicalize in the different languages. This is in line with other proposals which bind the variation in the lexicalization patterns to differences in the number of functional heads lexicalized by the adjectival/nominal/verbal root (see Caha et al. 2019; Vanden Wyngaerd et al. 2020 for adjectival morphology in Slavic; Caha et al. 2023 for adjectival/verbal *zero derivation* in English and Czech; Janků 2022 for Czech nominal declensions; Bertocci and Pinzin 2020 for verbal thematic vowels in Latin; Cortiula 2023 for verbal morphology in friulian). The nominal root in French and Emilian is constrained to the lexicalization of the [NP] denoting a property, as in (34), so that for all the subsequent features additional LIs are needed. Italian, instead, has bigger nominal roots, which can also lexicalize {indef} and the case features {f1}–{f2}, in addition to the [NP] predicative core. This is shown in (52c). The LIs for *def* and *DE* in (52a) and (52b) are the same as for the other languages.

(52)

**Table:** 

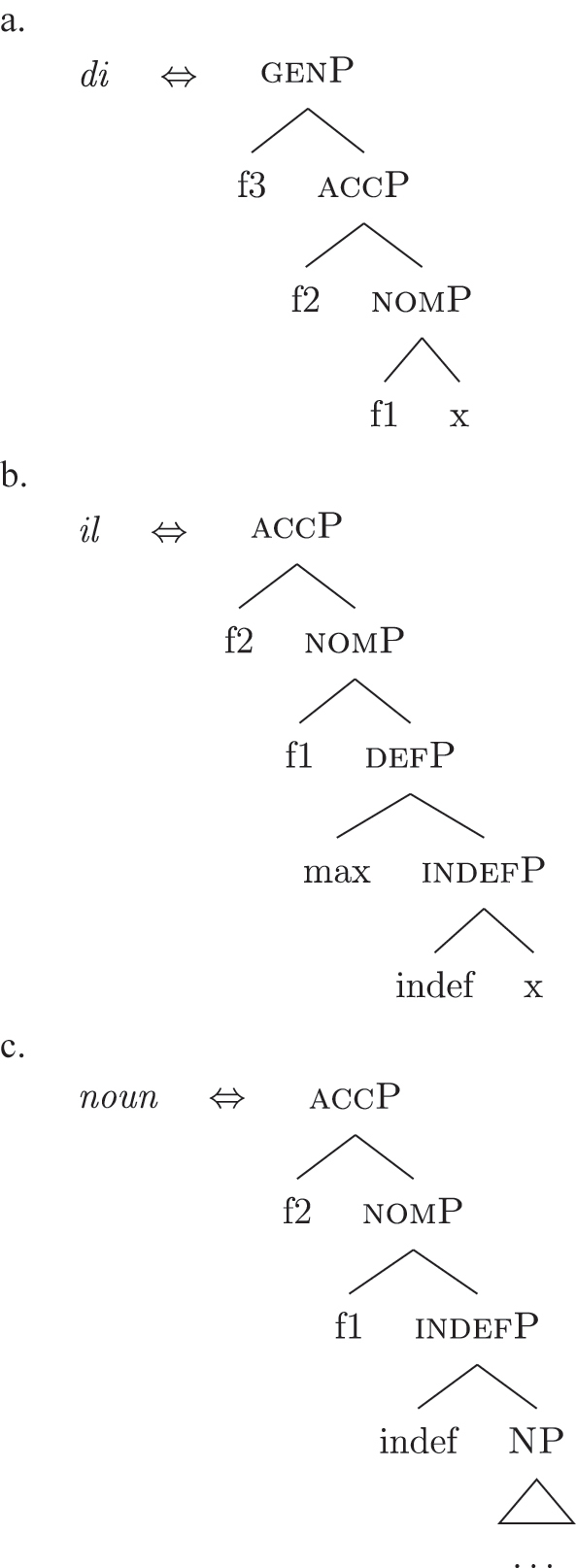

(52c) is a perfect match for the *fseq* in (33a) and, accordingly, fulfills step (a) of the lexicalization algorithm (direct lexicalization, with no movements) at each lexicalization cycle, outcompeting all other LIs. The result is shown in (53).

(53)

**Table:** 

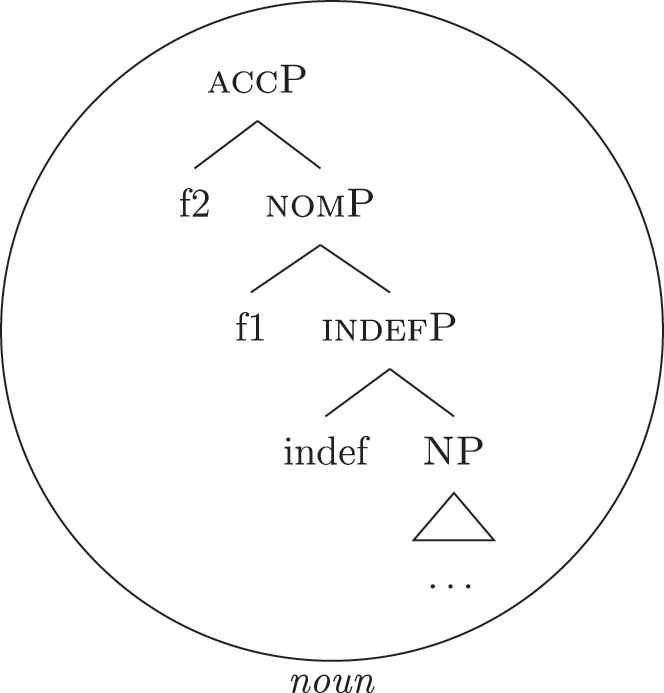

Note that (52c) can only be used if the syntactic derivation produces this exact constituent. In a definite derivation, {max} is merged with [indefP], yielding [defP]. Since (52c) does not contain [defP], it cannot lexicalize the derived constituent. In such a derivation, which I skip for reasons of space, the LI in (52a) must come into play, resulting in the syntactic configuration in (54).

(54)

**Table:** 

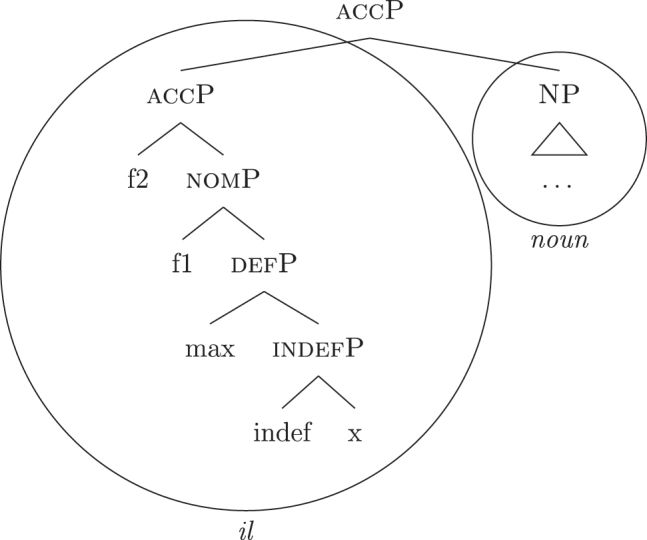

By the same logic, we can derive the Franco-Provençal pattern, which for unbounded indefinites has only *DE* and no *def* (see [13]). As for Italian, we keep constant all LIs but the one for the noun, which in Fr.Pr. stops lexicalizing at [indefP] and is therefore smaller than Italian but bigger than French/Emilian, as in (55).

(55)

**Table:** 

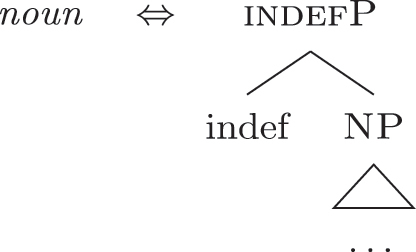

The resulting syntactic configuration is shown in (56), where *noun* and *de* lexicalize the whole structure.

(56)

**Table:** 

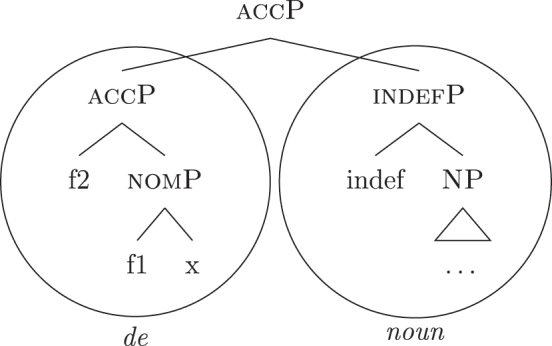

By allowing the number of functional heads that nominal roots can lexicalize to vary between languages, we derive the different patterns observed for unbounded indefinites (the first column of Table 1). The small nominal roots in French and Emilian (see [34c]) need both *def* and *DE* for lexicalizing the indefiniteness layer and the case layers, respectively; the slightly bigger nominal roots of the Franco-Provençal varieties (see [55]) only need *DE* for lexicalizing the case layers; finally, Italian has the biggest roots (see [52c]) and, accordingly, does not need other LIs.36In this and the following sections I will maintain that the size of the *noun* LI governs the different distribution of bounded/unbounded indefinites in these languages. An interesting issue is the role of the direct modifiers of the noun (e.g., adjectives) merged with the NP. Given their position, they should block the *noun* LI from lexicalizing higher portions of the structure (under constituent-based lexicalization, only the structurally highest LI can go on lexicalizing features). What happens in the higher layers should then depend on how much of the *fseq* adjectives can or cannot lexicalize. We could then expect a different behavior when nouns are preceded by adjectives, either in terms of favoring BNs or indefinite PAs. A reviewer rightly notes that Carlier and Lamiroy (2014) notice that prenominal adjectives disfavor the use of indefinite PAs, instead boosting the use of BNs. This is a potentially relevant fact, in that (some) adjectives might be taken to potentially lexicalize parts of the higher *fseq*, “bleeding” the use of PAs. The facts need, however, a careful review in each language before providing a potential modeling. On a more general level, this issue recalls the relation between adverbs and verbs: the presence/absence of adverbs does not affect how much of the verbal structure can be lexicalized by the verbal root itself or by additional suffixal markers or complex left branches/auxiliaries (see Starke 2021). Solving this issue requires a general approach to how adjunct-like elements are integrated in the *fseq*, which is beyond the scope of the current article. The LIs for *DE* and *def* are kept constant across languages.

### 5.2 Bounded indefinites: adding a second layer

If we take into consideration bounded indefinites (*I baked [some] bread*), we have a different crosslinguistic distribution of the LIs (see Table 1). More specifically, while French, Emilian, and Franco-Provençal keep the output constant (*DE* + *def*, *DE* + *def*, and *DE*, respectively), Italian differentiates bounded indefinites from the unbounded ones analyzed in the previous section, employing *DE* + *def* instead of BNs ([57]* *=* *[14]).

(57)

**Table:** 

*Ho*	*cotto*	*del*	*pane.* (It.)
have.1sg	baked	of.the.m.sg	bread
‘I baked (some) bread.’			

I propose that on the syntactic-semantic level bounded indefinites are more complex and that the difference can be formally encoded as an additional feature on top of {indef}. To identify this feature, let us focus on the different semantics of indefinite BNs and PAs in Italian. As already hinted in Section 1, I claim that indefinite PAs, in Italian, introduce a boundary to the amount/number of the element(s) having the property denoted by the noun. Let us consider the following examples in which we have the consumption verb *mangiare* ‘to eat’ in two different structures, one with the clitic pronoun *si* ‘self’ (the same clitic used in reflexive constructions) and one without. As independently supported in the literature, in these contexts the clitic *si* is resultative, meaning that it derives an event in which the object is consumed in its entirety (see Folli and Harley 2005).

(58)

a.

**Table:** 

*Da*	*qualche*	*mese,*	*Gianni*	*mangia*	*(del)*	*gelato*	*ogni*	*giorno* (It.)
from	some	month	Gianni	eats	of.the.m.sg	ice cream	every	day
‘It’s a few months that Gianni eats ice cream every day.’

b.

**Table:** 

*Da*	*qualche*	*mese,*	*Gianni*	*si*	*mangia*	*(del)	*gelato*	*ogni*
from	some	month	Gianni	self	eats	of.the.m.sg	ice cream	every
*giorno.* (It.)									
day								
‘It’s a few months that Gianni eats ice cream every day.’

While the possibility of using indefinite PAs is unaffected by the presence or absence of resultative *si*, the same is not true for BNs. BNs are ungrammatical in the context in which resultative *si* is present, as (58b) shows. These facts can be interpreted as following from the fact that (58b) requires the object to be delimited, to have boundaries, so that it is possible to consume it in its entirety. Notice, moreover, that we are not dealing with a requirement on the specificity of the object, given that both contexts in (58) denote a series of daily events in which the subject eats some ice cream each time, which cannot be the same specific ice cream. The ungrammaticality of the BN in (58b) can then be related to the fact that BNs lack a boundary, which is an essential semantic component of these structures, thus restricting the lexicalization possibilities of the object.37The situation partially changes with plural BNs, with some speakers accepting the sentence *Da qualche mese, Gianni si mangia gelati ogni giorno*. ‘It’s a few months that Gianni eats ice creams every day.’ If the interpretation I propose is correct, this would mean that for some speakers plural BNs can be interpreted as having a boundary/limit. One reviewer points out that this might be related to a kind of quantization introduced by the plural feature itself. While I agree with the interpretation, further data are needed to provide a complete analysis, especially with respect to the contrast between plural BNs and plural indefinite PAs. This also derives the incompatibility of Italian indefinite PAs with long-term habitual activity contexts like *I’ve been building houses for years*, where BNs are instead fully acceptable, as shown in (59a–b). In (59), the speaker is asserting that she has been doing the activity of building houses for years. The use of an indefinite PA in (59b) – which implies a boundary – makes it impossible to access this interpretation and instead forces an infelicitous one, in which the speaker asserts that she has been building an indefinite, bounded set of houses (specific or not) for years.

(59)

a.

**Table:** 

*Costruisco*	*case*	*da*	*anni.* (It.)
build.1sg	houses	from	years
‘I have been building houses for years.’

b.

**Table:** 

*Costruisco*	*delle*	*case*	*da*	*anni.* (It.)
build.1sg	of.the.f.pl	houses	for	years
#‘I have been building houses for years.’

Again, if we take indefinite PAs to be bounded, as opposed to BNs, we can explain this: the boundary imposes an interpretation of the object as an indefinite number of houses while the context requires an unbounded interpretation of the object as referring to any possible subset of the whole referent set of *case* ‘houses’.

Bearing these facts in mind, I label the feature differentiating these two types of indefinites as {boundary}, and the resulting phrase as [b(ounded)-indefP]. To reflect this formally, (60) presents the complete set of *fseqs*: unbounded indefinites are in (60a) and do not have the additional [b(ounded)-indefP] layer, while bounded indefinites and definites are in (60b) and (60c), respectively, and have the additional [b(ounded)-indefP] layer on top of [IndefP], hosting {boundary}. All *fseqs* are closed off by the case features, as has been argued for in Section 4. This highlights the different sizes of the gap: with respect to a definite *fseq*, unbounded indefinites lack two features in the middle ({boundary}, {max}), while bounded indefinites lack one feature ({max}).

(60)

**Table:** 

​ 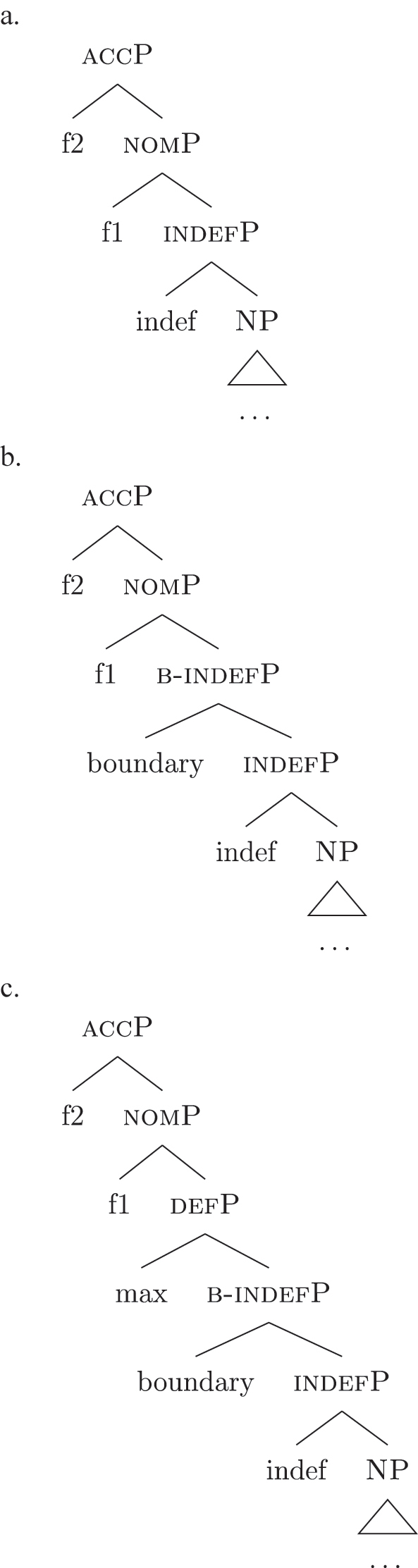

The facts regarding Italian, then, can be captured by updating the lexicon in (52) to the one in (61), where the only difference is the additional presence of [b-indefP] in (61b) (cfr. [52b], where no [b-indefP] is present). (52a) and (52c) remain intact.

(61)

**Table:** 

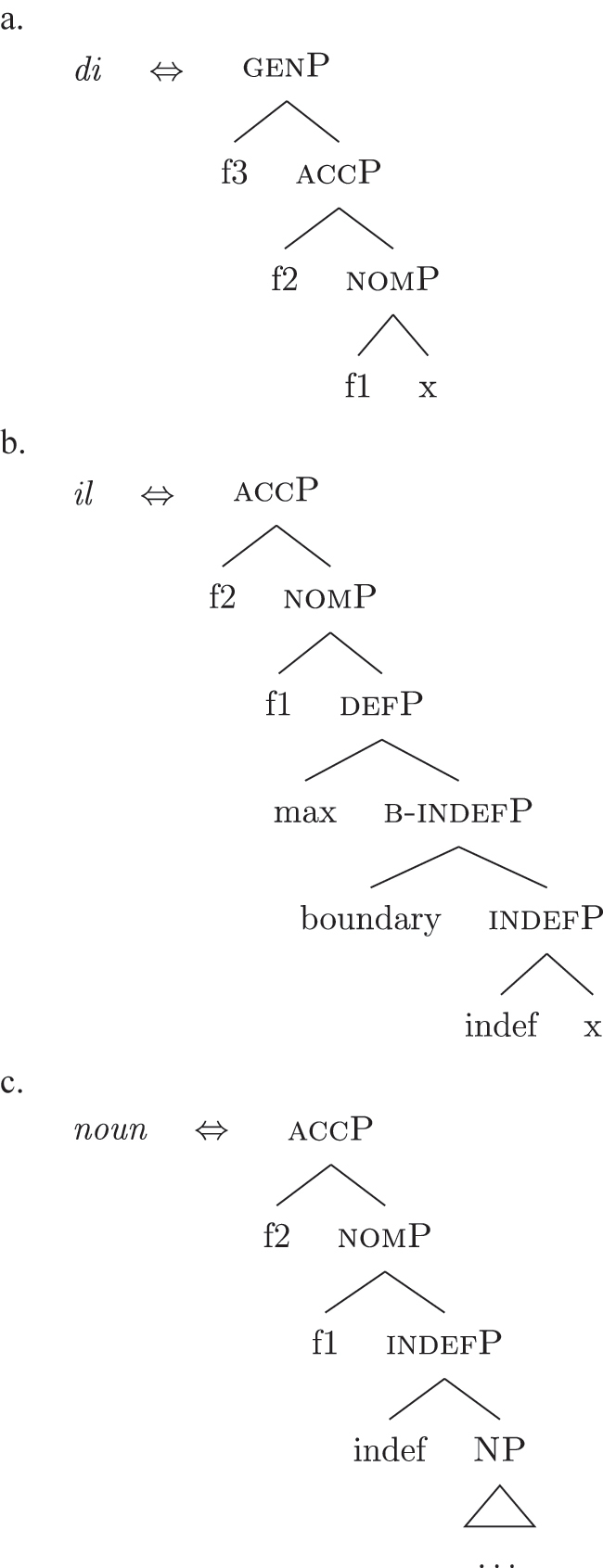

This lexicon, in conjunction with the lexicalization algorithm, derives the observed patterns. The *noun* LI in (61c) can lexicalize {indef} and the case features {f1}–{f2}, leading to the presence of BNs. This is only possible, however, when the *fseq* does not involve {boundary} or {max}, i.e., it is only possible with unbounded indefinites (60a). When {boundary} is merged and [b-indefP] is derived, we need to lexicalize it by means of the *def* LI *il* in (61b), which is the only one including the relevant feature. As shown before, then, if we are deriving a bounded indefinite (60b) – i.e., the *fseq* lacks {max} – the *DE* LI *di* in (61a) must be used to lexicalize {f1}–{f2}, yielding *di *+* il *+* noun* (62).

(62)

**Table:** 

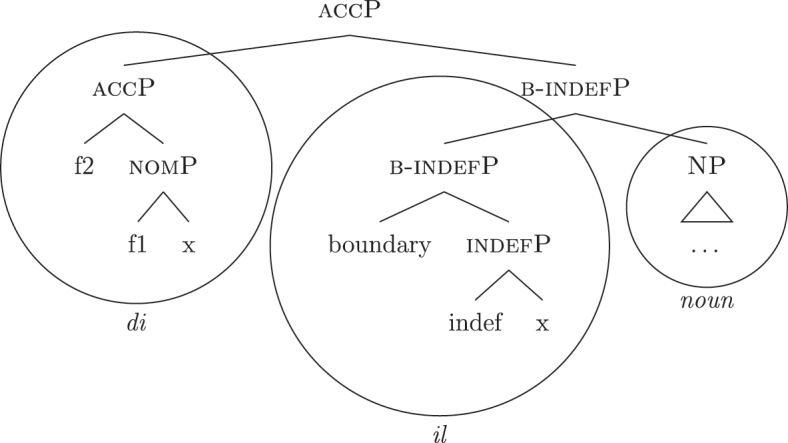

Note that the reason why the *DE* LI lexicalizes {f1}–{f2} in (62) instead of the *def* LI is the same as in (50) before (and [65] below): the relevant indefinite derivation contains a gap with respect to the definite derivation, i.e., it lacks {max}. As the *def* LI (61b) has {max} in between the indefiniteness layers and the case layers ({f1}–{f2}), it does not properly contain a constituent closed off by the case features but lacking {max}. By the Superset Principle, then, (61b) cannot lexicalize such constituent. If that is the case, *di* is inserted. By the same principles, if we are in a definite derivation – i.e., a derivation involving {max} – the LI *il* in (61b) can go on lexicalizing {f1}–{f2}, yielding *il *+* noun*.

As for the other varieties under analysis, we just need to update the LIs for accounting for the lexicalization of b-indefP. More specifically, for Emilian and French we must update the LI for *def* as we did for Italian (see [63b]), keeping the other LIs intact.

(63)

**Table:** 

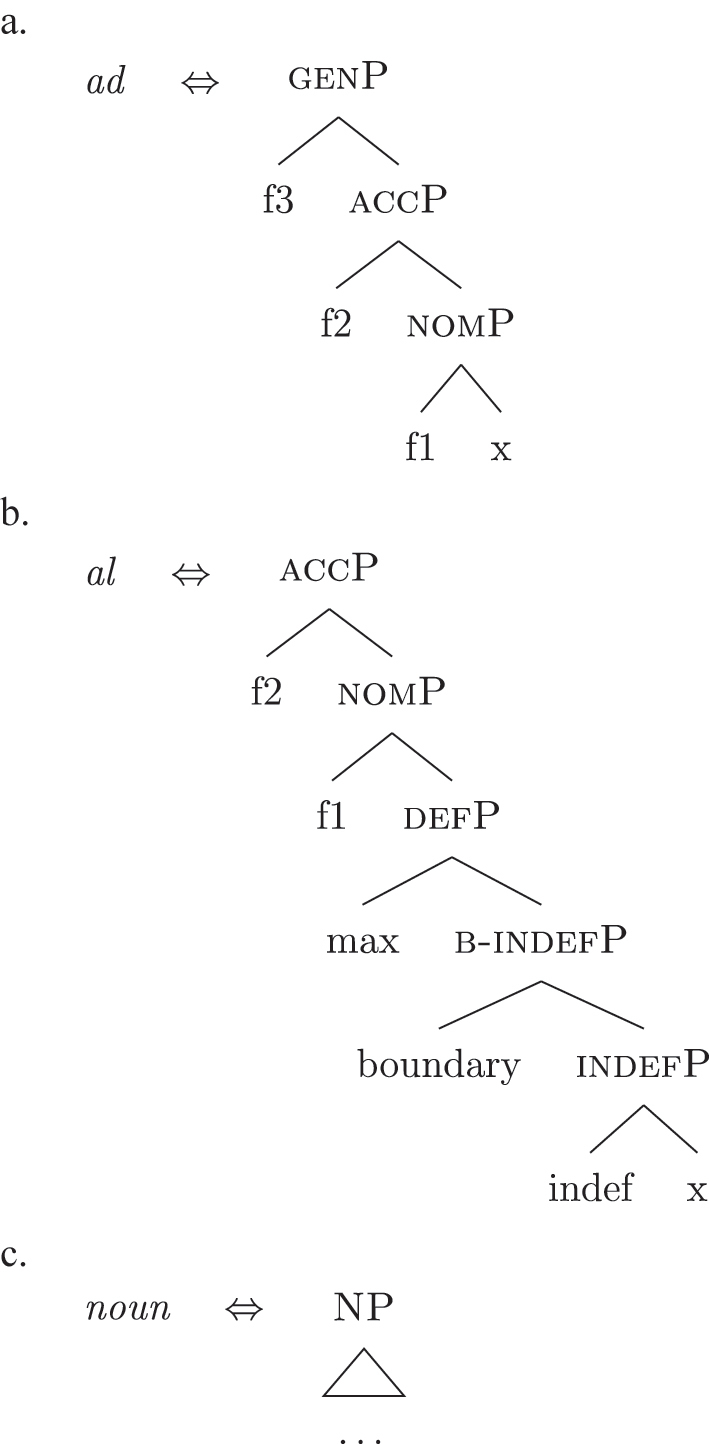

The *noun* LI is constrained to the [NP] level and, as in Italian, *def* can lexicalize {f1}–{f2} only if {max} is present. This yields *ad *+* al *+* noun* for both unbounded and bounded indefinites and *def *+* noun* for definites.

For Franco-Provençal, by contrast, we must update both the LI for *def*, as for the other varieties, and the *noun* LI, so that it can lexicalize {boundary} as in (64).

(64)

**Table:** 

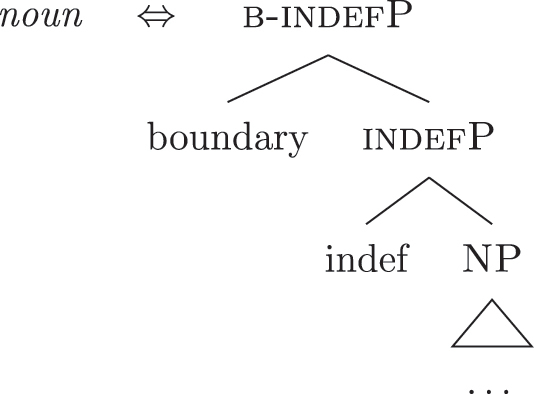

This yields *de *+* noun* for both the unbounded (60a) and the bounded (60b) indefinite *fseq* and *def *+* noun* for the definite *fseq* (60c). This follows from the fact that the *noun* LI can lexicalize every feature up to {boundary}, while {f1}–{f2} are always lexicalized by the *DE* LI as in (65), unless the feature {max} is merged.

(65)

**Table:** 

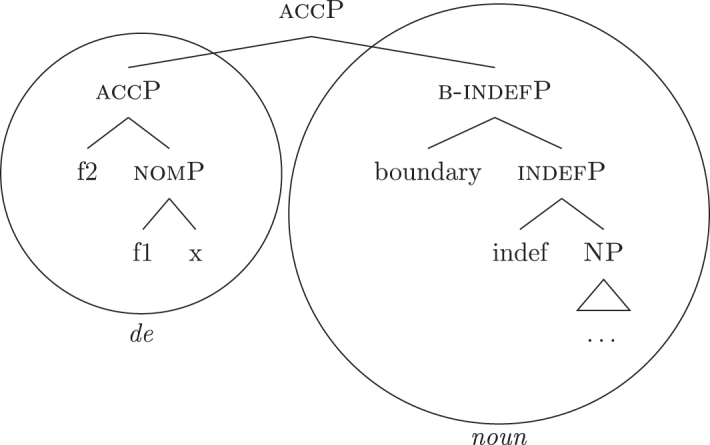

Note that at this point we derived the crosslinguistic variation shown in Table 1. The observed patterns reduce to how much of the *fseq* the noun can lexicalize in the different languages, in line with other current proposals (Bertocci and Pinzin 2020; Caha et al. 2019, 2023; Cortiula 2023; Janků 2022; Vanden Wyngaerd et al. 2020, among others). The LIs for *DE* and *def* remain crosslinguistically constant.

## 6 Splitting structural and oblique cases

In the previous sections, I only dealt with instances in which bounded and unbounded indefinites have structural case, that is nominative/accusative. Let us consider what happens in Italian in contexts in which non-structural cases are involved. I will illustrate this using data from Italian, but, as far as I can tell, this extends to all languages analyzed in this paper. In general, Italian introduces datives with the preposition *a* ‘to’. Keeping the noun constant (the proper name *Maria* in this case), *a* alone marks dative, while *di* alone marks genitive. No sequence *a* + *di* is attested.

(66)

**Table:** 

*Ho*	*dato*	*la*	*palla*	*a*	*Maria.* (It.)
have.1sg	given	the.f.sg	ball	to	Mary
‘I gave Mary the ball.’

(67)

**Table:** 

*La*	*palla*	*di*	*Maria.* (It.)
the.f.sg	ball	of	Mary
‘Mary’s ball.’			

Following, among others, Caha (2009, 2013, 2019, the set of case features following nominative/accusative directly builds on [accP], so that we have the *fseq* in (68).

(68)

**Table:** 

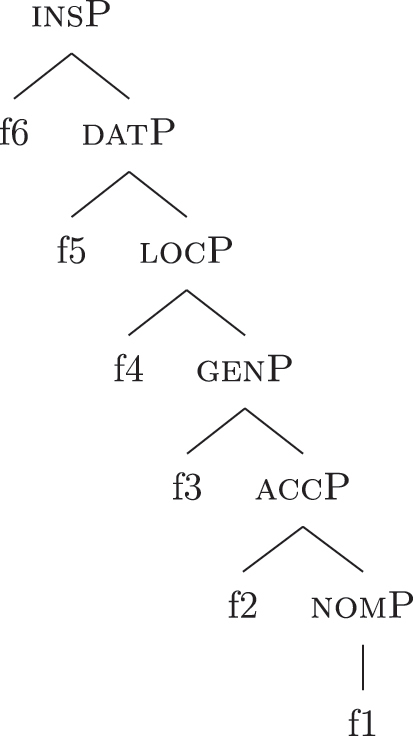

Dative is built on the genitive by adding {f4} and {f5}.38I leave aside the expression of locative for ease of exposition. However, note that in Italian pure locative (without any additional meaning, as, for example, being within a bounded space) is expressed syncretically with the dative as *a*.

(i)

**Table:** 

*Sono*	*a*	*casa/il supermercato.* (It.)
am	to	home/the supermarket.
‘I am at home/the supermarket.’

With this in place and within the adopted framework, the simplest analysis of the substitution pattern in (66)–(67) is to claim that the two markers do not co-occur because *di* (69a) is properly contained in *a* (69b).

(69)

**Table:** 

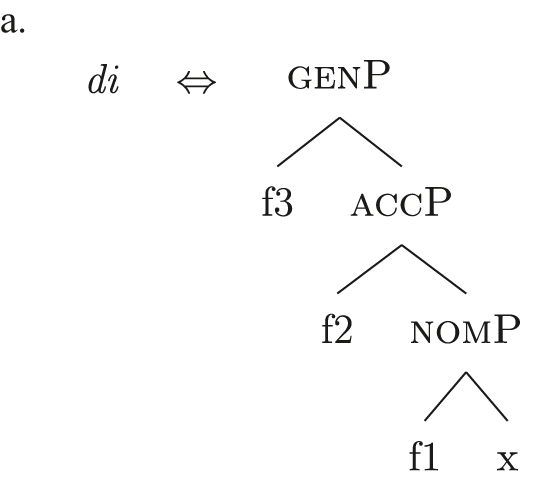
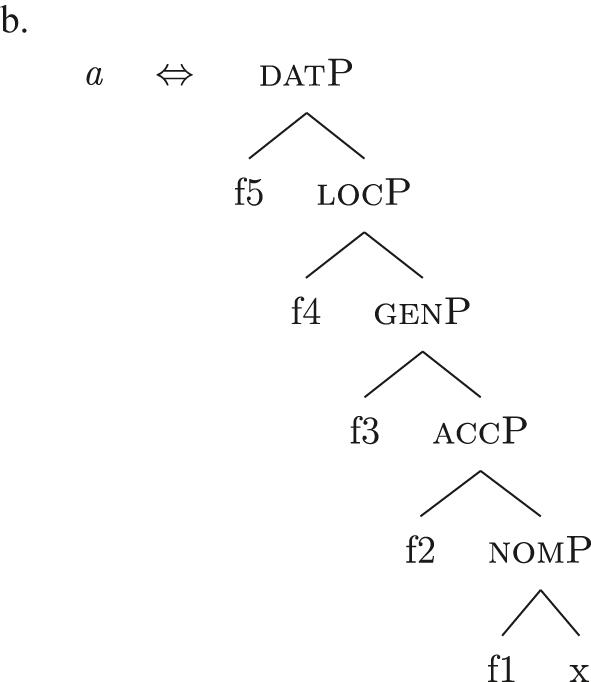

The LI *di*, being more specific, wins the competition up to [genP], while *a* wins as soon as we merge {f4} and {f5} and derive [datP], overriding *di*. This should extend to the use of *di* as an accusative/nominative marker too. In other words, assuming the case *fseq* in (68) and the LIs in (69), the prediction is that a dative bounded indefinite *fseq* should be lexicalized in Italian, as a sequence *a *+* def *+* noun* (i.e., with *a* overriding *di* for the lexicalization of the case *fseq*). This is shown in (70).

(70)

**Table:** 

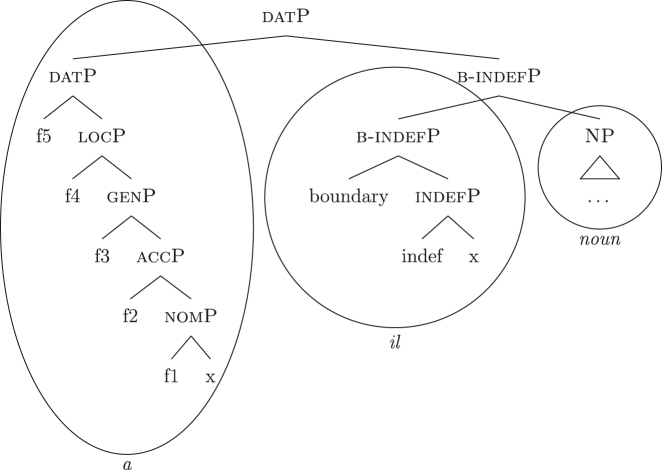

The prediction is wrong, however. *A* and *di* co-occur exactly in the indefinite contexts we are looking at, where we find the sequence *a* + *di*.39Note that there is nothing wrong with the sequence *a* + *il*, but it can only be interpreted as a dative introducing a definite argument, not as a dative introducing a bounded indefinite argument.

(i)

**Table:** 

*Ho*	*dato*	*la*	*palla*	*alle*	*ragazze.* (It.)
have.1sg	given	the.f.sg	ball	to.the.f.pl	girls
‘I gave the ball to the girls.’/*‘I gave the ball to some girls.’

(71)

**Table:** 

*Ho*	*dato*	*la*	*palla*	*a*	*delle*	*ragazze.* (It.)
have.1sg	given	the.f.sg	ball	to	of.the.f.pl	girls
‘I gave the ball to some girls.’						

This is not a peculiarity of *a*, since it happens with all other prepositions too: *da/con/per delle ragazze* ‘from/with/for of.the.f.pl (= some) girls’ (see Carlier 2007; Ihsane 2008 for similar remarks on French).40Some Italian speakers, while accepting all other prepositions introducing an indefinite PA, do not fully accept *di*. For example, an indefinite PA in the complement of *parlare di* ‘talk about’ (whose complement is introduced by the P *di* ‘of’: *io parlo di te* ‘I talk about you’) is judged suboptimal.

(i)

**Table:** 

^??^ *Parlo*	*di*	*dei*	*ragazzi.* (It.)
speak.1sg	of	of.the.m.pl	boys
‘I am talking about some boys.’

A search of the Italian corpus of the web on SketchEngine (ItTenTen20, Jakubicek et al. 2013) gives 4,643 hits for the sequence *di delle* ‘of.the.f.pl’ (as of December 2023).

(ii)

**Table:** 

*Il PTPR si compone* ***di delle*** *norme tecniche di attuazione, nonché di delle tavole grafiche.* (It.)
‘The PTPR is composed by some (= of of.the.f.pl) technical regulations for implementation, as well by some graphical tables.

Given the size of the corpus (>14 billion tokens at the time of consulting it for this paper), this testifies that this is a minority pattern. It also testifies, however, that at least some speakers produce this kind of sentences some of the time. In any case, what is relevant for the argumentation is that no “simplification” is attested in these contexts. Even if marginal, the only way to combine a verb like *parlare di* ‘talk about’ with an indefinite PA is by the sequence *di* + *di* + *def*. More simply, *di* co-occurs with these other prepositions only in those contexts in which I claim that it lexicalizes a structural case, either nominative or accusative. There is then a split between how “structural” and “genitive” *di* behave. In principle, this issue can be dealt with either by modifying the LIs or the *fseq*. One approach could be to revive the homophony idea, claiming that we have two homophonous LIs *di*, one specified for nominative/accusative (72b) and the other for genitive (72a). The LI for *a* – (72c) – would then contain only (72a), “genitive” *di*.

(72)

**Table:** 

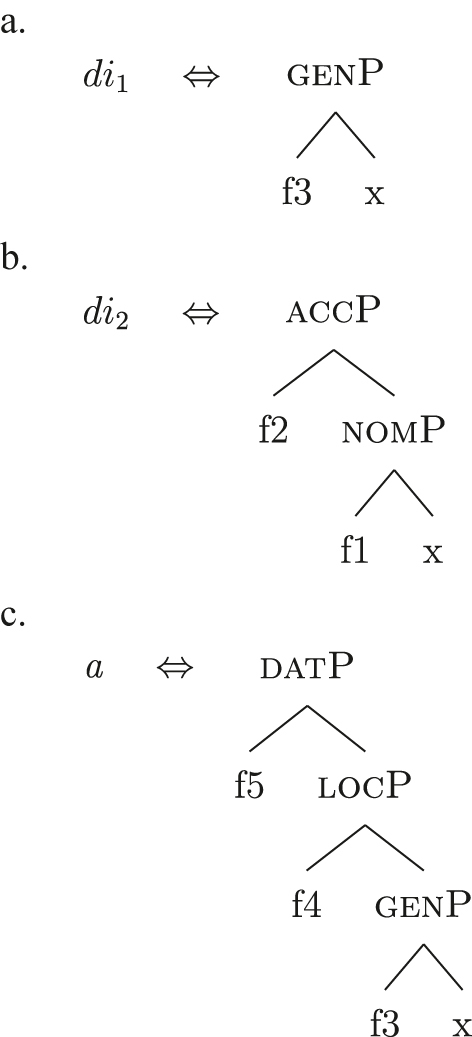

This would superficially fix the problem, at the cost of giving up the idea I put forward in this contribution, that is, to adopt a unique lexical entry for *DE* (and *def*) in all its uses, proposed at the outset of the paper as an alternative to the homophony approach that makes use of at least two LIs for *DE*. Moreover, note that by (72a–b) we would have two homophonous LIs lexicalizing contiguous pieces of the *fseq*. To avoid this outcome, I propose modifying the *fseq* instead. The observed pattern splits “structural” *di* from “oblique” *di*. Let us then hypothesize that the case *fseq*, in these cases at least, is not contiguous as in (68) but split in two, with an additional feature – here labeled {z} – intervening between accusative and the rest of the case sequence, as shown in (73).

(73)

**Table:** 

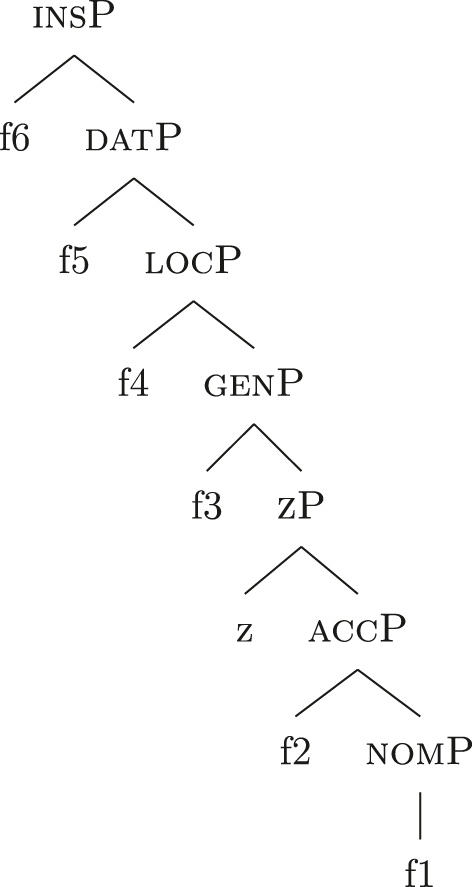

Let us further assume that the only LI that contains {z} in the lexicon is a phonologically null LI with a complex foot (i.e., a “pre”-marker).

(74)

**Table:** 

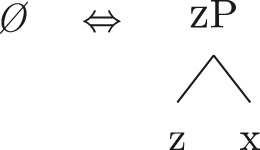

In this scenario, the LI in (74) would block any interaction between the higher part of the case *fseq* and the lower part. It follows that there is no stage of the derivation in which the higher case features ({f3}, {f4}, etc.) are merged directly on top of the *pre*-marker *di* lexicalizing [accP]. As a result, *a* would not be able to overwrite “structural” *di*.

(75)

**Table:** 

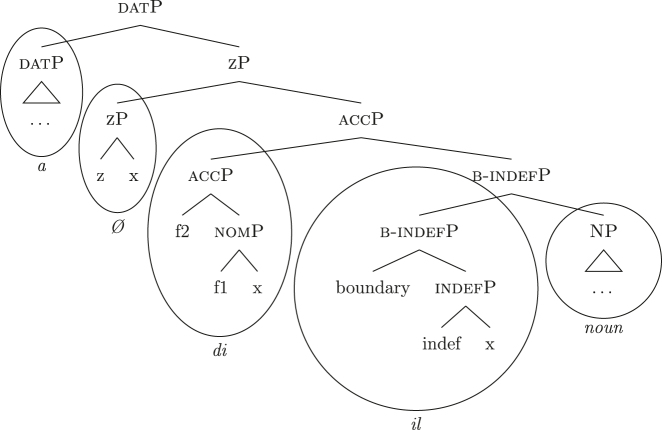

While {z} would shield an occurrence of “structural” *di* from being overwritten by *a*, the same would not happen if *di* is merged above it (i.e., it is an “oblique” *di*), since in that case the higher case features would be directly merged on top of the pre-marker *di*. Following this hypothesis, one could maintain a single *di* LI and explain the different patterns occurring when it lexicalizes structural and oblique cases.41As a reviewer points out, this approach is reminiscent of the “scattered heads” proposal by Giorgi and Pianesi (1997). In their framework, a LI x can project a sequence of features on a single terminal, unless the initial array of LIs at the basis of the specific derivation contains a LI y projecting a feature in the middle of that sequence; in such case, the features of LI x are scattered on different terminals and movement between such terminals is enabled. Notwithstanding the differences in terms of the order of operations and the basic units of computation, the approach in Giorgi and Pianesi (1997) takes crosslinguistic variation as stemming from the number of features connected to the LIs of each language, making it compatible with the current proposal.

An evident drawback of this proposal is that it requires stipulating an *ad hoc* null pre-marker in the lexicon, which would satisfy an unspecified feature {z}.42Note, however, that proposing an intervening head in the *fseq* for case is coherent with the fact that structural and non-structural cases have been found to behave in a consistently asymmetric way in different languages (see Bayer et al. 2001; Czypionka et al. 2018 for German, and references therein). However, the stipulation of a null *pre*-marker is not needed *a priori*. It could be possible to achieve the same effect by assuming that {z} is lexicalized by a feature-driven movement of a constituent from within its complement [accP]. This displaced constituent would block the interaction between the “structural” and the “oblique” layer as much as a phonologically null *pre*-element. Since there is still no full-fledged proposal incorporating feature-driven movement into the lexicalization algorithm in the literature (but see De Clercq 2020 for a potential approach) and developing one is beyond the scope of this paper, we set this issue aside for future work.

## 7 Conclusions

In this paper I put forward a novel proposal regarding the morphosyntactic structure of indefinite partitive articles in Romance languages and, from a broader perspective, why we find what otherwise appears to be a genitive preposition (*DE*) in nominative/accusative contexts and what otherwise appears to be a definite article (*def*) in indefinite contexts. In doing so, I maintain that the lexicon of these languages contains a single LI for each of *DE* and *def*. Note that, within the boundaries of the present data, this fact holds crosslinguistically too: the lexically stored tree for the LI *DE* is identical in each language analyzed, and the same is valid for *def*. The core of the proposal is that the *DE* and *def* LIs contain, respectively, nominative-accusative ({f1}–{f2}) and indefinite ({indef}-{boundary}) features, in addition to the features characterizing them as genitive ({f3}) and definite ({max}). This is exemplified in (76a–b) with the LIs for the Mantovano Emilian variety.

(76)

**Table:** 

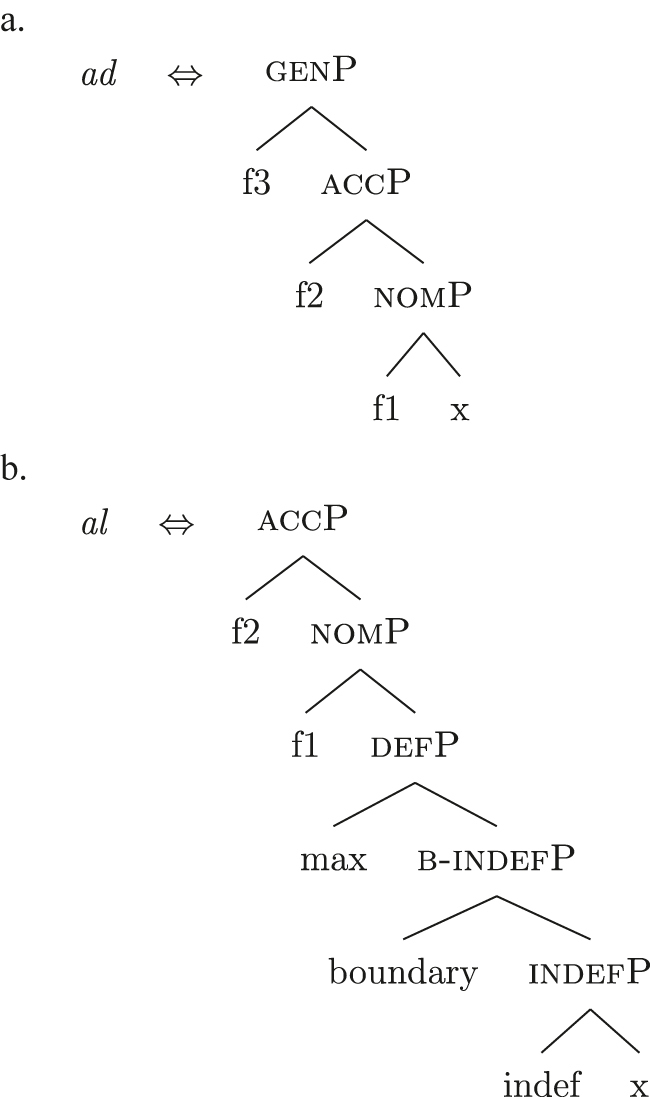

The fact that in French and Emilian the two types of indefinite *fseqs* analyzed – unbounded and bounded indefinites (Section 5.2) – come out as a sequence of *DE *+* def* while definite *fseqs* just as *def* is a consequence of constituent lexicalization. The LI *def* can lexicalize the {f1}–{f2} case features only if it also lexicalizes the definiteness feature {max}. This means that a definite *fseq* with structural case will surface as *def* alone, without any additional marker. If, on the other hand, {max} is not merged in the *fseq* (i.e., we have an indefinite *fseq*, unbounded or bounded), the LI *DE* wins the competition for the lexicalization of {f1}–{f2}, yielding the sequence *DE *+* def*. In the paper, I also highlighted how different Romance languages vary with respect to how they lexicalize unbounded and bounded indefinites (Section 2). I propose that this crosslinguistic variation can be accounted for in terms of the “size” of the nominal root, that is the number of functional features that it can lexicalize. French and Emilian have the “smallest” nominal roots, confined to lexicalizing the property [NP] (63c). This results in both bounded and unbounded indefinites being lexicalized as *DE *+* def *+* noun*. Some other languages, such as some Franco-Provençal varieties, have nominal roots growing to [b-indefP] (64). Such roots are therefore able to lexicalize both layers of indefiniteness but need the LI *DE* to lexicalize nominative/accusative case features, yielding *DE *+* noun* for both bounded and unbounded indefinites. Finally, languages such as Italian have nominal roots growing up to nominative/accusative case features but only incorporating the features for lexicalizing the layer for unbounded indefinites (61c). This results in a surface pattern in which we see just the noun (BN) as a lexicalization for unbounded indefinites and *DE *+* def *+* noun* as a lexicalization of bounded of indefinites.

## Supplementary Material

Supplementary Material
