# Supplementary material for: What’s hidden below definiteness and genitive: on indefinite partitive articles in Romance
Source: Linguistics. 2024 Apr 18;62(5):1251–300. doi: 10.1515/ling-2022-0059 (PMC11382604; doi:10.1515/ling-2022-0059)

$di_1 \Leftrightarrow$

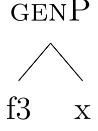

Supplement: Supplementary file 1 — Supplementary Material [file j_ling-2022-0059_suppl_001.zip › trees_submission/Linguistics_trees (60).pdf]
