# Supplementary material for: What’s hidden below definiteness and genitive: on indefinite partitive articles in Romance
Source: Linguistics. 2024 Apr 18;62(5):1251–300. doi: 10.1515/ling-2022-0059 (PMC11382604; doi:10.1515/ling-2022-0059)
Supplement: Supplementary file 1 — Supplementary Material [file j_ling-2022-0059_suppl_001.zip › trees_submission/Linguistics_trees (30).pdf]

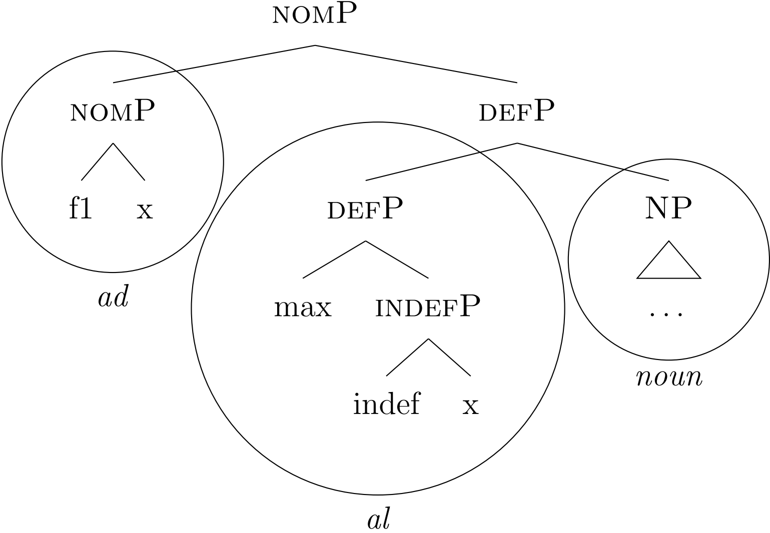
NOMP
NOMP
f1
x
ad
DEFP
DEFP
max
INDEFP
indef
x
al
NP
...
noun
