# Supplementary figures and images for: What’s hidden below definiteness and genitive: on indefinite partitive articles in Romance
Source: Linguistics. 2024 Apr 18;62(5):1251–300. doi: 10.1515/ling-2022-0059 (PMC11382604; doi:10.1515/ling-2022-0059)

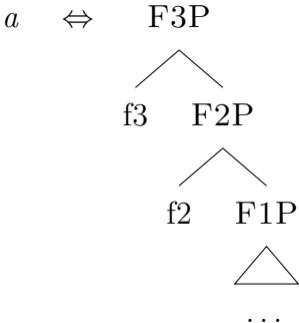

Supplement: Supplementary file 1 — Supplementary Material [file j_ling-2022-0059_suppl_001.zip › trees_submission/Linguistics_trees (7).pdf]

ACCP

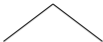

f2

NOMP

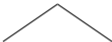

f1

INDEFP

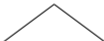

indef

NP

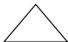

...

Supplement: Supplementary file 1 — Supplementary Material [file j_ling-2022-0059_suppl_001.zip › trees_submission/Linguistics_trees (17).pdf]

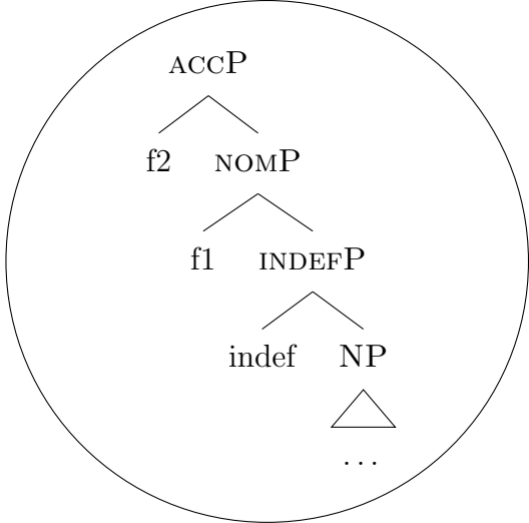

*noun*

Supplement: Supplementary file 1 — Supplementary Material [file j_ling-2022-0059_suppl_001.zip › trees_submission/Linguistics_trees (40).pdf]

INSP

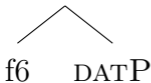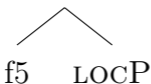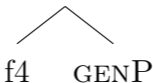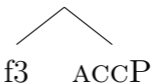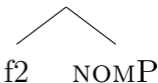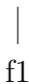

Supplement: Supplementary file 1 — Supplementary Material [file j_ling-2022-0059_suppl_001.zip › trees_submission/Linguistics_trees (56).pdf]

*noun*

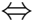

NP

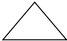

...

Supplement: Supplementary file 1 — Supplementary Material [file j_ling-2022-0059_suppl_001.zip › trees_submission/Linguistics_trees (21).pdf]

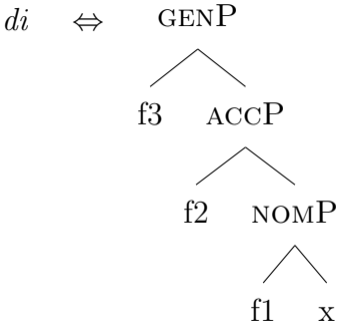

Supplement: Supplementary file 1 — Supplementary Material [file j_ling-2022-0059_suppl_001.zip › trees_submission/Linguistics_trees (37).pdf]

$di_2$

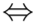

ACCP

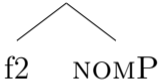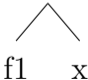

Supplement: Supplementary file 1 — Supplementary Material [file j_ling-2022-0059_suppl_001.zip › trees_submission/Linguistics_trees (61).pdf]

NOMP

NOMP

f1 x

*ad*

INDEFP

INDEFP

indef x

*al*

NP

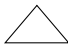

...

*noun*

Supplement: Supplementary file 1 — Supplementary Material [file j_ling-2022-0059_suppl_001.zip › trees_submission/Linguistics_trees (36).pdf]

*al*

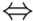

DEFP

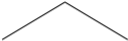

max

INDEF P

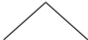

indef

x

Supplement: Supplementary file 1 — Supplementary Material [file j_ling-2022-0059_suppl_001.zip › trees_submission/Linguistics_trees (20).pdf]

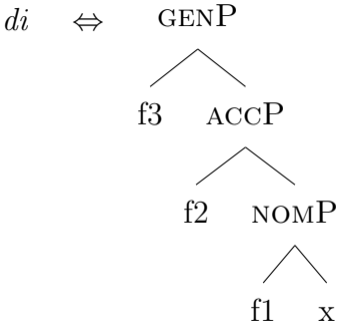

Supplement: Supplementary file 1 — Supplementary Material [file j_ling-2022-0059_suppl_001.zip › trees_submission/Linguistics_trees (57).pdf]

ACCP

ACCP

f2

NOMP

f1

DEFP

max

INDEFP

indef

x

NP

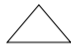

...

*noun*

*il*

Supplement: Supplementary file 1 — Supplementary Material [file j_ling-2022-0059_suppl_001.zip › trees_submission/Linguistics_trees (41).pdf]

*pre*

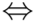

F2P

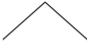

f2

F1P

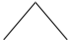

f1

x

Supplement: Supplementary file 1 — Supplementary Material [file j_ling-2022-0059_suppl_001.zip › trees_submission/Linguistics_trees (16).pdf]

F3P

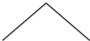

f3

F2P

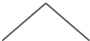

f2

F1P

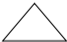

...

Supplement: Supplementary file 1 — Supplementary Material [file j_ling-2022-0059_suppl_001.zip › trees_submission/Linguistics_trees (6).pdf]

NOMP

NOMP

f1 x

*ad*

INDEFP

INDEFP

indef x

*al*

NP

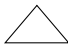

...

*noun*

Supplement: Supplementary file 1 — Supplementary Material [file j_ling-2022-0059_suppl_001.zip › trees_submission/Linguistics_trees (27).pdf]

$\emptyset$  $\Leftrightarrow$ 

ACCP

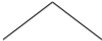

f2

NOMP

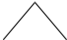

f1

x

Supplement: Supplementary file 1 — Supplementary Material [file j_ling-2022-0059_suppl_001.zip › trees_submission/Linguistics_trees (31).pdf]

*ad*

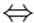

GENP

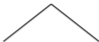

f3

ACCP

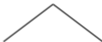

f2

NOMP

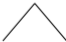

f1

x

Supplement: Supplementary file 1 — Supplementary Material [file j_ling-2022-0059_suppl_001.zip › trees_submission/Linguistics_trees (66).pdf]

CONST3

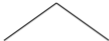

*z*

CONST2

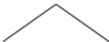

*y*

CONST3

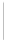

*x*

Supplement: Supplementary file 1 — Supplementary Material [file j_ling-2022-0059_suppl_001.zip › trees_submission/Linguistics_trees (1).pdf]

$a \iff \text{F3P}$

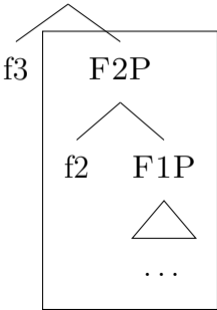

Supplement: Supplementary file 1 — Supplementary Material [file j_ling-2022-0059_suppl_001.zip › trees_submission/Linguistics_trees (11).pdf]

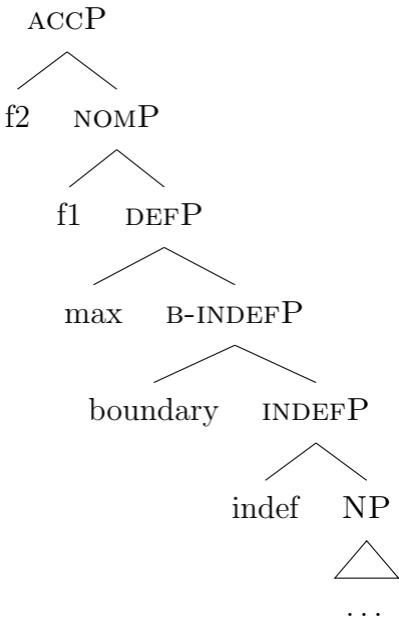

Supplement: Supplementary file 1 — Supplementary Material [file j_ling-2022-0059_suppl_001.zip › trees_submission/Linguistics_trees (46).pdf]

ACCP

B-INDEFP

ACCP

f2

NOMP

f1

x

B-INDEFP

boundary

INDEFP

indef

x

NP

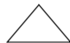

...

*noun*

*di*

*il*

Supplement: Supplementary file 1 — Supplementary Material [file j_ling-2022-0059_suppl_001.zip › trees_submission/Linguistics_trees (50).pdf]

*ad*

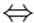

GENP

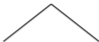

f3

ACCP

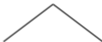

f2

NOMP

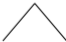

f1

x

Supplement: Supplementary file 1 — Supplementary Material [file j_ling-2022-0059_suppl_001.zip › trees_submission/Linguistics_trees (51).pdf]

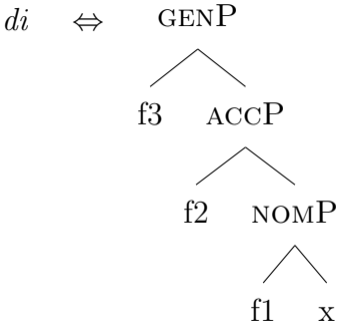

Supplement: Supplementary file 1 — Supplementary Material [file j_ling-2022-0059_suppl_001.zip › trees_submission/Linguistics_trees (47).pdf]

F2P

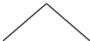

f2

F1P

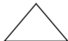

...

*a*

Supplement: Supplementary file 1 — Supplementary Material [file j_ling-2022-0059_suppl_001.zip › trees_submission/Linguistics_trees (10).pdf]

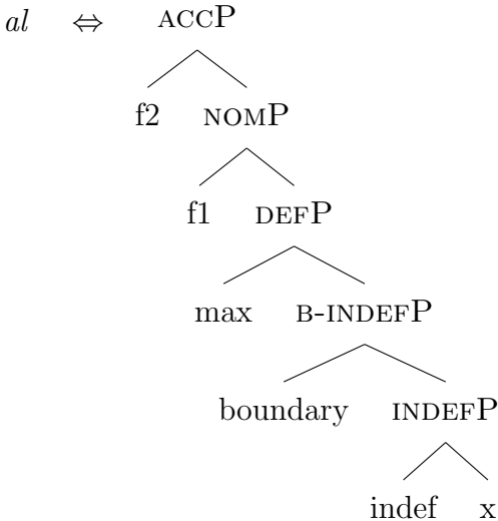

Supplement: Supplementary file 1 — Supplementary Material [file j_ling-2022-0059_suppl_001.zip › trees_submission/Linguistics_trees (67).pdf]

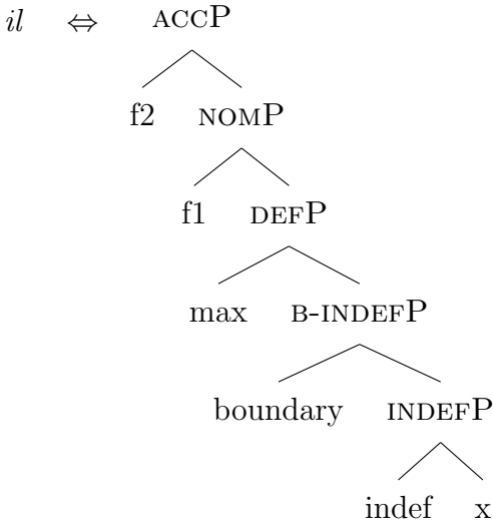

Supplement: Supplementary file 1 — Supplementary Material [file j_ling-2022-0059_suppl_001.zip › trees_submission/Linguistics_trees (48).pdf]

$\emptyset$

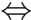

$ZP$

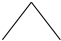

$Z$

$X$

Supplement: Supplementary file 1 — Supplementary Material [file j_ling-2022-0059_suppl_001.zip › trees_submission/Linguistics_trees (64).pdf]

ACCP

ACCP

f2

NOMP

f1

DEFP

max

INDEFP

indef

x

NP

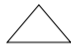

...

*noun*

*al*

Supplement: Supplementary file 1 — Supplementary Material [file j_ling-2022-0059_suppl_001.zip › trees_submission/Linguistics_trees (33).pdf]

NOMP

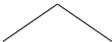

f1 INDEFP

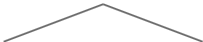

INDEFP

NP

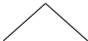

indef x

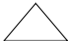

...

Supplement: Supplementary file 1 — Supplementary Material [file j_ling-2022-0059_suppl_001.zip › trees_submission/Linguistics_trees (25).pdf]

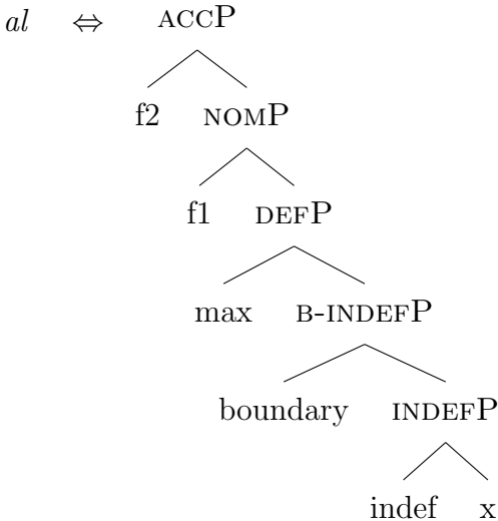

Supplement: Supplementary file 1 — Supplementary Material [file j_ling-2022-0059_suppl_001.zip › trees_submission/Linguistics_trees (52).pdf]

ACCP

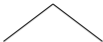

f2

NOMP

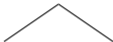

f1

INDEFP

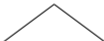

indef

NP

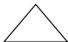

...

Supplement: Supplementary file 1 — Supplementary Material [file j_ling-2022-0059_suppl_001.zip › trees_submission/Linguistics_trees (44).pdf]

$a \iff \text{F3P}$

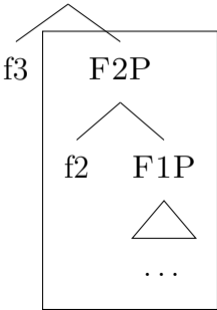

Supplement: Supplementary file 1 — Supplementary Material [file j_ling-2022-0059_suppl_001.zip › trees_submission/Linguistics_trees (13).pdf]

DEFP

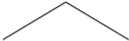

max

INDEFP

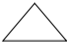

...

Supplement: Supplementary file 1 — Supplementary Material [file j_ling-2022-0059_suppl_001.zip › trees_submission/Linguistics_trees (3).pdf]

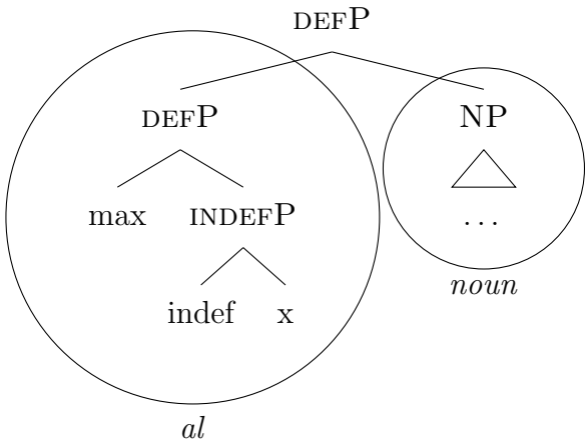

Supplement: Supplementary file 1 — Supplementary Material [file j_ling-2022-0059_suppl_001.zip › trees_submission/Linguistics_trees (29).pdf]

DEFP

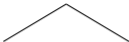

max

INDEFP

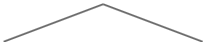

INDEFP

NP

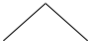

indef

x

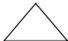

...

Supplement: Supplementary file 1 — Supplementary Material [file j_ling-2022-0059_suppl_001.zip › trees_submission/Linguistics_trees (28).pdf]

GENP

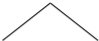

f3

ACCP

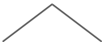

f2

NOMP

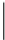

f1

Supplement: Supplementary file 1 — Supplementary Material [file j_ling-2022-0059_suppl_001.zip › trees_submission/Linguistics_trees (2).pdf]

F2P

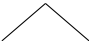

f2

F1P

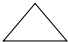

...

*b*

Supplement: Supplementary file 1 — Supplementary Material [file j_ling-2022-0059_suppl_001.zip › trees_submission/Linguistics_trees (12).pdf]

ACCP

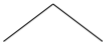

f2

NOMP

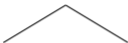

f1

B-INDEFP

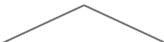

boundary

INDEFP

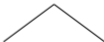

indef

NP

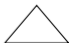

...

Supplement: Supplementary file 1 — Supplementary Material [file j_ling-2022-0059_suppl_001.zip › trees_submission/Linguistics_trees (45).pdf]

*noun*

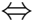

NP

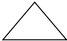

...

Supplement: Supplementary file 1 — Supplementary Material [file j_ling-2022-0059_suppl_001.zip › trees_submission/Linguistics_trees (53).pdf]

ACCP

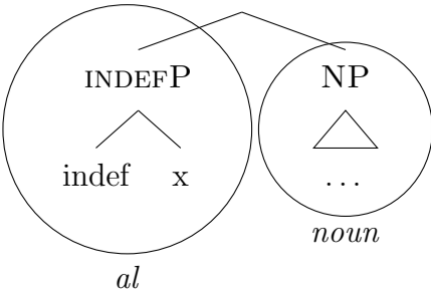

Supplement: Supplementary file 1 — Supplementary Material [file j_ling-2022-0059_suppl_001.zip › trees_submission/Linguistics_trees (24).pdf]

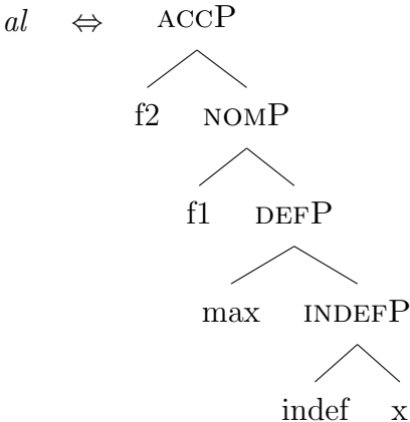

Supplement: Supplementary file 1 — Supplementary Material [file j_ling-2022-0059_suppl_001.zip › trees_submission/Linguistics_trees (32).pdf]

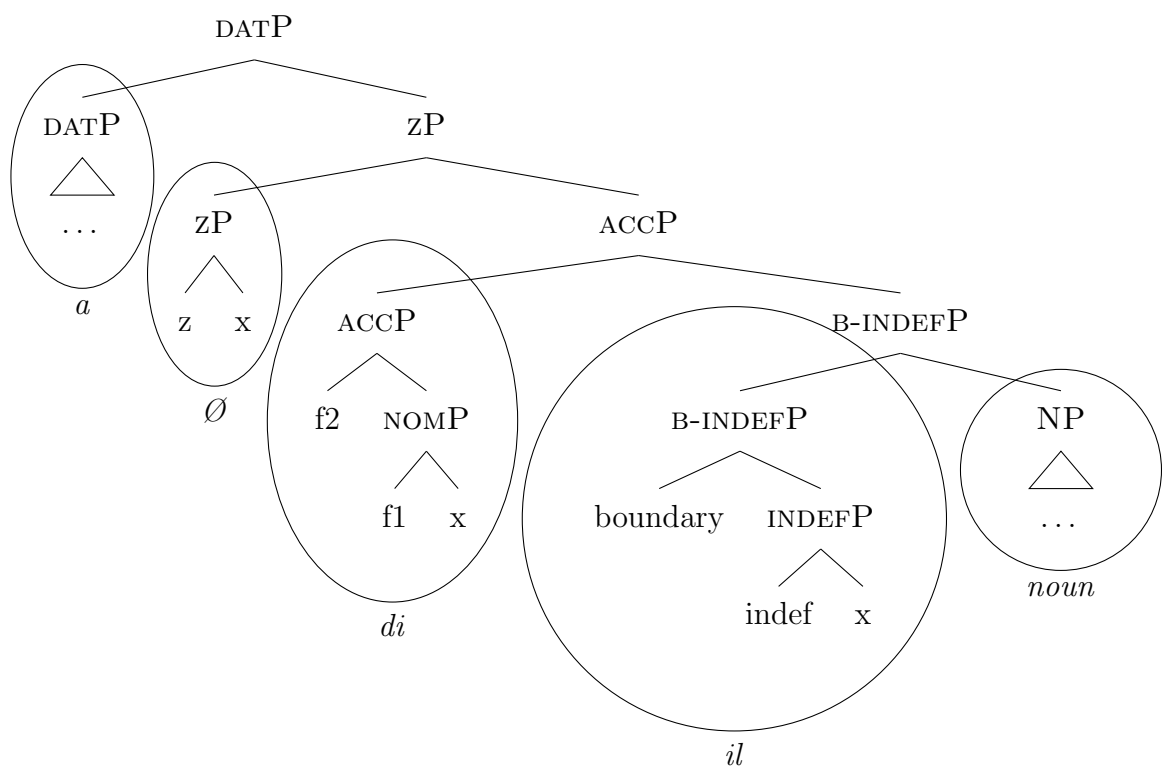

Supplement: Supplementary file 1 — Supplementary Material [file j_ling-2022-0059_suppl_001.zip › trees_submission/Linguistics_trees (65).pdf]

*noun*     $\Leftrightarrow$

ACCP

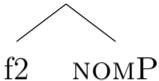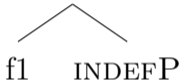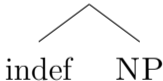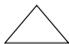

...

Supplement: Supplementary file 1 — Supplementary Material [file j_ling-2022-0059_suppl_001.zip › trees_submission/Linguistics_trees (49).pdf]

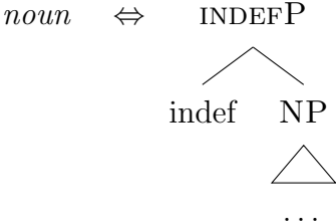

Supplement: Supplementary file 1 — Supplementary Material [file j_ling-2022-0059_suppl_001.zip › trees_submission/Linguistics_trees (42).pdf]

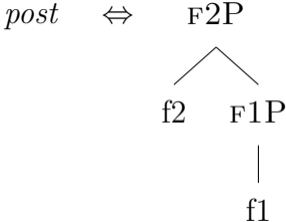

Supplement: Supplementary file 1 — Supplementary Material [file j_ling-2022-0059_suppl_001.zip › trees_submission/Linguistics_trees (15).pdf]

NOMP

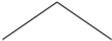

f1

INDEFP

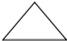

...

Supplement: Supplementary file 1 — Supplementary Material [file j_ling-2022-0059_suppl_001.zip › trees_submission/Linguistics_trees (5).pdf]

*noun*     $\Leftrightarrow$

ACCP

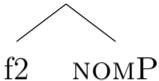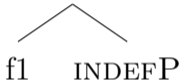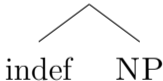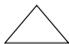

...

Supplement: Supplementary file 1 — Supplementary Material [file j_ling-2022-0059_suppl_001.zip › trees_submission/Linguistics_trees (39).pdf]

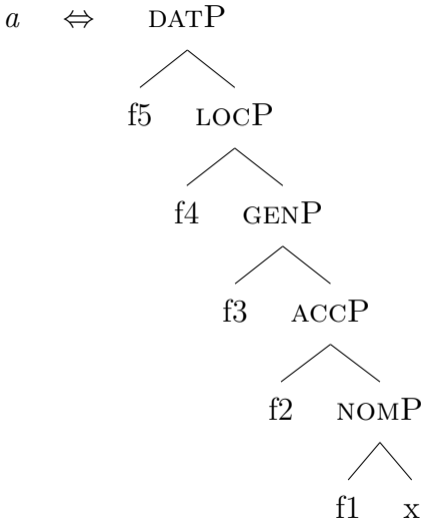

Supplement: Supplementary file 1 — Supplementary Material [file j_ling-2022-0059_suppl_001.zip › trees_submission/Linguistics_trees (58).pdf]

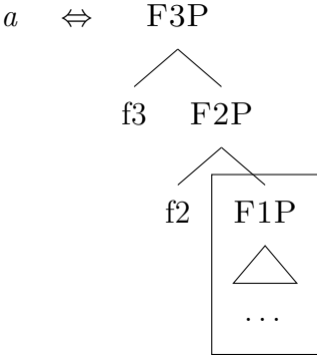

Supplement: Supplementary file 1 — Supplementary Material [file j_ling-2022-0059_suppl_001.zip › trees_submission/Linguistics_trees (9).pdf]

*ad*

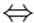

GENP

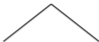

f3

ACCP

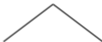

f2

NOMP

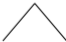

f1

x

Supplement: Supplementary file 1 — Supplementary Material [file j_ling-2022-0059_suppl_001.zip › trees_submission/Linguistics_trees (19).pdf]

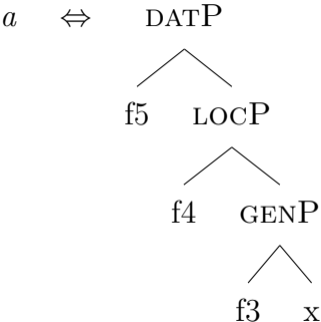

Supplement: Supplementary file 1 — Supplementary Material [file j_ling-2022-0059_suppl_001.zip › trees_submission/Linguistics_trees (62).pdf]

INDEFP

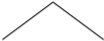

indef

NP

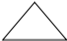

...

Supplement: Supplementary file 1 — Supplementary Material [file j_ling-2022-0059_suppl_001.zip › trees_submission/Linguistics_trees (23).pdf]

NP

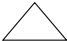

...

*noun*

Supplement: Supplementary file 1 — Supplementary Material [file j_ling-2022-0059_suppl_001.zip › trees_submission/Linguistics_trees (22).pdf]

NOMP

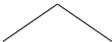

f1

INDEFP

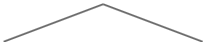

INDEFP

NP

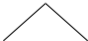

indef

x

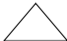

...

Supplement: Supplementary file 1 — Supplementary Material [file j_ling-2022-0059_suppl_001.zip › trees_submission/Linguistics_trees (34).pdf]

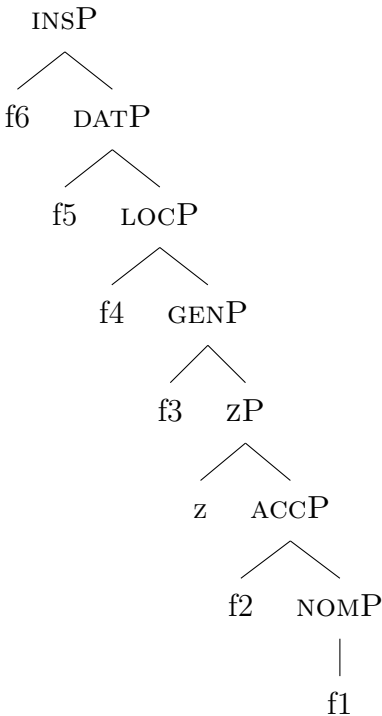

Supplement: Supplementary file 1 — Supplementary Material [file j_ling-2022-0059_suppl_001.zip › trees_submission/Linguistics_trees (63).pdf]

ACCP

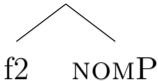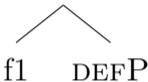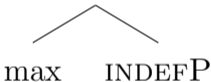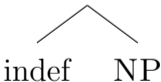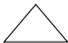

...

Supplement: Supplementary file 1 — Supplementary Material [file j_ling-2022-0059_suppl_001.zip › trees_submission/Linguistics_trees (18).pdf]

F1P

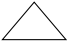

...

*a*

Supplement: Supplementary file 1 — Supplementary Material [file j_ling-2022-0059_suppl_001.zip › trees_submission/Linguistics_trees (8).pdf]

DATP

DATP

f5

LOCP

f4

GENP

f3

ACCP

f2

NOMP

f1

x

*a*

B-INDEFP

B-INDEFP

boundary

INDEFP

indef

x

*il*

NP

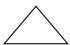

...

*noun*

Supplement: Supplementary file 1 — Supplementary Material [file j_ling-2022-0059_suppl_001.zip › trees_submission/Linguistics_trees (59).pdf]

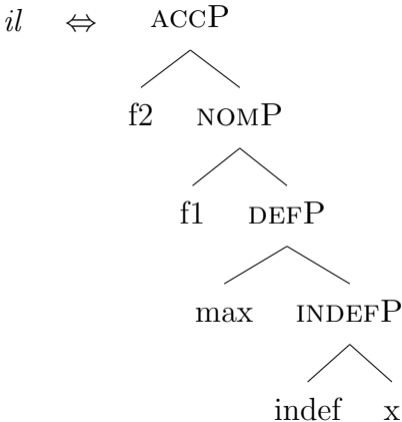

Supplement: Supplementary file 1 — Supplementary Material [file j_ling-2022-0059_suppl_001.zip › trees_submission/Linguistics_trees (38).pdf]

NOMP

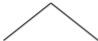

f1

DEFP

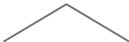

max

INDEFP

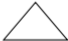

...

Supplement: Supplementary file 1 — Supplementary Material [file j_ling-2022-0059_suppl_001.zip › trees_submission/Linguistics_trees (4).pdf]

$b$

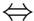

F2P

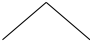

f2

F1P

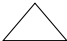

...

Supplement: Supplementary file 1 — Supplementary Material [file j_ling-2022-0059_suppl_001.zip › trees_submission/Linguistics_trees (14).pdf]

ACCP

ACCP

f2

NOMP

f1

x

*de*

INDEF P

indef

NP

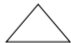

...

*noun*

Supplement: Supplementary file 1 — Supplementary Material [file j_ling-2022-0059_suppl_001.zip › trees_submission/Linguistics_trees (43).pdf]

ACC*P*

ACC*P*

f2

NOM*P*

f1

x

*de*

B-INDEF*P*

boundary

INDEF*P*

indef

NP

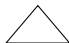

...

*noun*

Supplement: Supplementary file 1 — Supplementary Material [file j_ling-2022-0059_suppl_001.zip › trees_submission/Linguistics_trees (55).pdf]
